# Combatting Antibiotic-Resistant *Staphylococcus aureus*: Discovery of TST1N-224, a Potent Inhibitor Targeting Response Regulator VraRC, through Pharmacophore-Based Screening and Molecular Characterizations

**DOI:** 10.1021/acs.jcim.4c01046

**Published:** 2024-07-30

**Authors:** Ying-Chu Hsu, Ching-Hui Liu, Yi-Chen Wu, Shu-Jung Lai, Chi-Jan Lin, Tien-Sheng Tseng

**Affiliations:** † Division of Neurology, Department of Internal Medicine, Ditmanson Medical Foundation ChiaYi Christian Hospital, Chiayi 600566, Taiwan; ‡ Institute of Molecular Biology, National Chung Hsing University, Taichung 40202, Taiwan; § Graduate Institute of Biomedical Sciences, China Medical University, Taichung 404333, Taiwan; ∥ Research Center for Cancer Biology, China Medical University, Taichung 404333, Taiwan

## Abstract

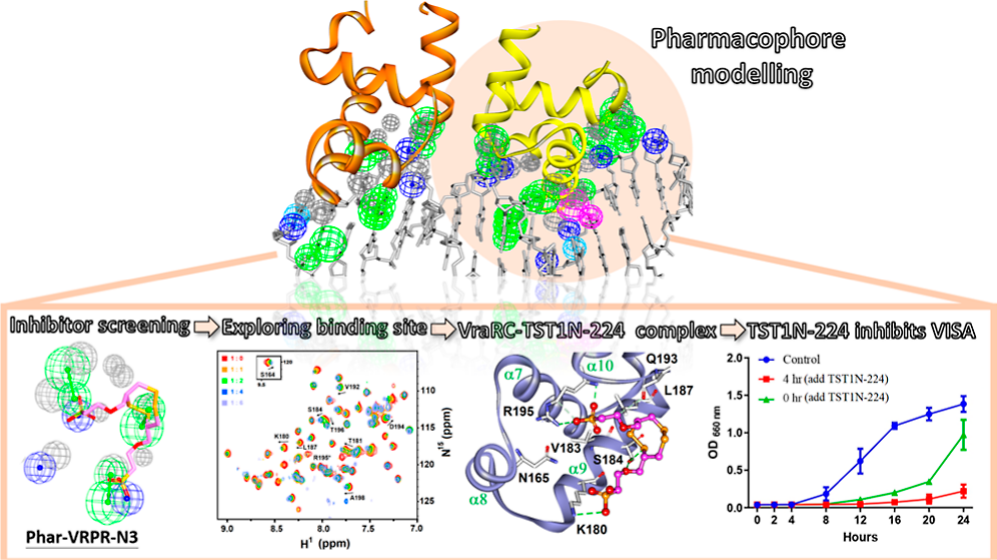

*Staphylococcus aureus* (*S. aureus*) is a major global health concern, causing various infections and presenting challenges due to antibiotic resistance. In particular, methicillin-resistant *S. aureus*, vancomycin-intermediate *S. aureus* (VISA), and vancomycin-resistant *S. aureus* pose significant obstacles in treating *S. aureus* infections. Therefore, the critical need for novel drugs to counter these resistant forms is pressing. Two-component systems (TCSs), integral to bacterial regulation, offer promising targets for disruption. In this study, a comprehensive approach, involving pharmacophore-based inhibitor screening, along with biochemical and biophysical analyses were conducted to identify, characterize, and validate potential inhibitors targeting the response regulator VraRC of *S. aureus*. The constructed pharmacophore model, **Phar-VRPR-N3**, demonstrated effectiveness in identifying a potent inhibitor, **TST1N-224** (IC_50_ = 60.2 ± 4.0 μM), against the formation of the VraRC-DNA complex. Notably, **TST1N-224** exhibited strong binding to VraRC (KD = 23.4 ± 1.2 μM) using a fast-on-fast-off binding mechanism. Additionally, NMR-based molecular modeling revealed that **TST1N-224** predominantly interacts with the α9- and α10-helixes of the DNA-binding domain of VraR, where the interactive and functionally essential residues (N165, K180, S184, and R195) act as hotspots for structure-based inhibitor optimization. Furthermore, **TST1N-224** evidently enhanced the susceptibility of VISA to both vancomycin and methicillin. Importantly, **TST1N-224** distinguished by 1,2,5,6-tetrathiocane with the 3 and 8 positions modified with ethanesulfonates holds significant potential as a lead compound for the development of new antimicrobial agents.

## Introduction

More than a century ago, *Staphylococcus aureus*, a prominent human pathogen, was identified as the primary cause of various infections.^1^ These infections vary from relatively minor cases like suppurative abscesses and soft tissue infections to severe and life-threatening conditions such as chronic osteomyelitis, pneumonia, endocarditis, and other illnesses associated with significant mortality and morbidity.^2^ In the mid-20th century, antibiotics such as methicillin and penicillin were initially effective against *S. aureus*. However, the rapid development of resistance in *S. aureus* gave rise to the emergence of methicillin-resistant *S. aureus* (MRSA).^3^ In the late 1990s, MRSA strains resistant to multiple drugs became the predominant causative agents of *S. aureus* infections, occurring in both community and hospital settings.^4^ During this period, it is crucial to highlight that vancomycin, a glycopeptide antibiotic, remained the primary treatment for MRSA infections.^5^ Vancomycin is a frequently utilized antibiotic in hospital environments to address severe infections caused by MRSA strains.^6^ Its mode of action entails binding to a specific cellular component called lipid II dipeptide d-Ala4-d-Ala5 to disrupt crucial biological processes.^7,8^ These processes include transglycosylation, transpeptidation, and peptidoglycan modification, which are typically orchestrated by enzymes like PBP2 and PBP2a.^9,10^ Commencing in 1980, the prevalent use of vancomycin was propelled by the growing frequency of MRSA infections in hospitals worldwide.^11^ However, the escalating dependence on vancomycin gave rise to vancomycin-resistant *S. aureus* (VRSA) strains. In 1977, Japan reported *S. aureus* strains with reduced vancomycin susceptibility, and later, complete vancomycin resistance in VRSA strains was discovered.^12^

The VRSA and VISA are strains of antibiotic-resistant *S. aureus*.^13^ VRSA stands for vancomycin-resistant *S. aureus*. The infections of VRSA are particularly concerning, as they are resistant to vancomycin, the last-resort antibiotic for treating severe MRSA infections.^14^ VRSA infections have been associated with higher mortality rates compared to nonresistant strains of MRSA, and treatment options for VRSA infections are limited.^15^ Likewise, VISA stands for vancomycin-intermediate *S. aureus* and refers to strains of MRSA that are less susceptible to vancomycin.^4,16,17^ VISA infections are also concerning, as they are less susceptible to vancomycin, indicating higher doses or longer treatment periods are required to achieve the desired therapeutic effect.^18^ This led to prolonged hospitalization and increased healthcare costs, and in some cases, treatment may not be effective, resulting in a higher risk of mortality. Both VRSA and VISA pose significant challenges for treating MRSA infections, as they limit the effectiveness of vancomycin and other antibiotics traditionally used to treat these infections.^19^ The mortality due to VRSA and VISA is influenced by the infection severity, patient health, and treatment availability. These antibiotic-resistant *S. aureus* strains are widely recognized as a significant public health threat. The increase in the level of VRSA and VISA emphasizes the urgent need for ongoing research and the development of new antibiotics and alternative treatments to combat antibiotic-resistant bacteria and prevent their spread. Importantly, understanding the mechanisms of antibiotic resistance by which this system operates could lead to the development of new strategies for treating *S. aureus* infections and combating antibiotic-resistant bacteria.

One of the mechanisms contributing to stress response and bacterial adaptation is a two-component signal transduction system (TCS).^20^ The TCS is an important mechanism by which bacteria can respond to changes in their environment, including exposure to antibiotics.^21^ The TCS involves a conserved phosphotransfer pathway between a histidine kinase (HK) and its response regulator (RR).^22^ It is initiated when the HK senses an environmental signal and autophosphorylates, generating a phosphoryl group that is transferred to a conserved aspartate residue on the receiver domain (RD) of the RR.^23^ The RR typically contains a RD and a DNA-binding domain (DBD) connected by a linker region. This phosphorylation event activates the RR, leading to a conformational change that can enhance its binding to DNA and increase its activity as a transcription factor.^24^ This, in turn, can lead to changes in gene expression and cellular metabolism that allow the bacteria to adapt to its environment. In *S. aureus*, the vancomycin resistance-associated sensor/regulator (VraSR) two-component signal transduction system is involved in sensing and responding to cell wall stress caused by vancomycin exposure.^25^ The VraSR system consists of VraS (HK) and VraR (RR). When activated by cell wall stress, VraS phosphorylates VraR, which in turn binds to specific DNA sequences to regulate gene expression.^26^ Specifically, VraR belongs to the NarL/FixJ family of response regulators, which are characterized by a helix-turn-helix (HTH) DNA-binding domain at their C-terminus.^27^ This domain allows VraR to bind to specific DNA sequences and regulate the expression of genes involved in virulence and other cellular processes. In addition to its role in vancomycin resistance, the VraSR system has also been shown to play a role in regulating virulence in *S. aureus*.^28^ Accordingly, the VraSR TCS is an important regulatory system in *S. aureus* that plays a role in both antibiotic resistance and virulence. Thus, the development of inhibitors targeting VraSR is a feasible strategy to disrupt the ability of VRSA and VISA to adapt and survive in the presence of vancomycin.

Structurally, VraR undergoes phosphorylation-induced conformational changes, transitioning from a closed monomeric state to an open dimeric form that facilitates DNA binding.^27^ This regulates downstream gene expression, impacting cell wall synthesis, and other cellular processes. Additionally, the crystal structure of VraRC in complex with R1-DNA highlights potent binding interactions, featuring a compact dimer between DNA-binding domains.^29^ The positively charged surface of the VraRC complements the negatively charged DNA phosphodiester backbone. Also, it has been suggested that interfering with VraR binding to cognate DNA could potentially disrupt the ability of *S. aureus* to respond to cell wall stress and thereby offer a novel target for the development of new antibiotics.^27^ Therefore, the availability of structures of VraR along and in complex with DNA makes it feasible to screen and develop potent inhibitors by structure-based and computer-aided drug design (CADD). In this study, we utilized the structural information on the VraRC-DNA complex to conduct pharmacophore-based inhibitor screening. This approach, combined with biochemical and biophysical analyses, allowed us to identify, characterize, and validate potential inhibitors targeting VraRC. The constructed pharmacophore model, **Phar-VRPR-N3**, comprised essential DNA-binding features, facilitating the mapping of ligands for the screening of potential inhibitors. The top 10 ranked hits identified through ligand-pharmacophore mapping from the IBS database were subjected to inhibition assays. As a result, two compounds displayed 50% inhibition against the formation of the PhoP-DNA complex at a concentration of 100 μM. Subsequent assays demonstrated that **TST1N-224** (IC_50_ = 60.2 ± 4.0 μM) and **TST1N-691** (IC_50_ = 75.2 ± 6.2 μM) displayed dose-dependent inhibitions, disrupting the formation of the VraRC-DNA complex. As well, localized surface plasmon resonance (LSPR) investigations revealed a strong binding of **TST1N-224** to VraRC (KD = 23.4 ± 1.2 μM). Additionally, NMR-based molecular modeling elucidated the mode of action of **TST1N-224** against VraRC. Moreover, the in vitro antibacterial assays elucidated the efficacy of **TST1N-224** against VISA. This integrated approach, combining CADD with biochemical and biophysical techniques, successfully identified, characterized, and validated the inhibitor **TST1N-224**, specifically targeting VraRC of VISA. **TST1N-224** is of great potential for further optimization into therapeutic agents combating drug-resistant *S. aureus*.

## Materials and Methods

### Preparations of the Recombinant VraRC Protein

The C-terminal DNA binding domain of the VraR (VraRC; residues 138-209) gene was cloned into the pET-GB1 vector using Nde I and Xho I restriction sites. Additionally, a C-terminal His-tag was introduced to facilitate protein purification. The VraRC plasmid was transformed into *E. coli* BL21(DE3) for overexpression. The bacteria were cultured in LB medium at 37 °C with 50 mg/L kanamycin. When the cell density reached an optical density of OD_600_ = 0.6, the cells were induced with 0.6 mM IPTG (isopropyl β-D-1-thiogalactopyranoside) and grown for an additional 4 h. Subsequently, the cultured cells were harvested by centrifugation at 6000 rpm for 20 min. The resulting cell pellet was then lysed using a microfluidizer in a lysis buffer containing 20 mM Tris–HCl (pH 8.0), 500 mM NaCl, and 2% glycerol. The supernatant obtained from the crude extract was subjected to purification using a nickel-nitrilotriacetic acid (Ni-NTA) affinity resin (Qiagen, Hilden, Germany). Furthermore, the concentrated protein solution was then passed through a size exclusion column (Superdex 75 10/300) for purification. For ^15^N-labeled VraRC protein, the cells were grown in M9 minimal medium containing ^15^NH_4_Cl (1 g/L) at 37 °C and further induced, overexpressed, and purified with the same procedures mentioned above. The purity of the protein sample was assessed using a Coomassie blue-stained sodium dodecyl-sulfate (SDS) polyacrylamide gel. The bicinchoninic acid assay (BCA) method was employed to determine the concentration of the protein using bovine serum albumin as a standard.

### Preparations of DNA Fragments

The double-stranded oligonucleotides (5′-AGACTAAAGTATGAACATCATT-3′ and 3′-TCTGATTTCATACTTGTAGTAA-5′) used for the biophysical study of VraRC^29^ were synthesized and purchased from Yao-Hong Biotechnology Inc. To generate the double-stranded DNA (dsDNA), equal aliquots of the two oligomers were mixed in a 20 mM sodium phosphate buffer and 150 mM NaCl solution (pH 6.0). The mixture was then heated to 95 °C and slowly cooled to room temperature, allowing the oligomers to anneal and form a double-stranded structure. To purify the annealed dsDNA products, ion exchange chromatography was employed using a Mono-Q 5/50 GL column (Amersham Biosciences).

### Analyses of DNA Binding Properties of VraRC by Fluorescence Polarization Assay

The oligonucleotide (5′-AGACTAAAGTATGAACATCATT-3′) used for the fluorescence polarization experiment was labeled with 6-carboxyfluorescein (6-FAM) at the 5′ positions. Different concentrations of the VraRC protein were added to wells of an ELISA plate containing 10 nM of the 6-FAM-labeled DNA in a reaction buffer consisting of 20 mM sodium phosphate and 150 mM NaCl at pH 6.0. The reactions were carried out at 25 °C for 10 min. Measurement of the reactions was performed using a Synergy H1MF plate reader (BioTek Instruments, Inc.). The plate reader was set with an excitation wavelength of 485 nm and an emission wavelength of 535 nm, and the reactions were measured three times to obtain reliable data. The obtained data were analyzed by using GraphPad Prism 6 software (San Diego, CA, USA). Binding curves were fitted to one- or two-binding models to determine the binding affinity and kinetics of the VraRC protein to DNA.

### Receptor–Ligand Pharmacophore Generation and Pharmacophore-Based Inhibitor Screening (Ligand Pharmacophore Mapping)

To identify the functionally important features necessary for ligands to interact with target proteins, we employed receptor–ligand pharmacophore modeling. Specifically, we utilized the complex structure of the VraRC-DNA complex (PDB ID: 7VE5) to build the pharmacophore model. The pharmacophore model was generated using the receptor–ligand pharmacophore generation module of Discovery Studio 2021 (Accelrys Software, Inc., San Diego, CA, USA). The VraRC structure was designated as the “Input Receptor”, while the DNA structure was used as the “Input Ligand”. The parameters were set as follows: “Minimum Features” and “Maximum Features” were set to 10 and 30, respectively, and the maximum number of pharmacophores was set to 10. For conformation generation, the “fast method” with the “rigid fitting method” was applied. Default settings were used for the remaining parameters. Subsequently, the generated pharmacophore model was employed for ligand-pharmacophore mapping, which screened potential inhibitors based on the pharmacophore features. All 68,000 compounds from the IBS database (https://www.ibscreen.com/) were fitted to the pharmacophore model using the “flexible” fitting method. The remaining parameters were kept at the default settings.

### Inhibitory Activities of Compounds Determined by Fluorescence Polarization Measurements

The oligonucleotide (5′-AGACTAAAGTATGAACATCATT-3′)^29^ was labeled with 6-carboxyfluorescein (6-FAM) at the 5′ position and dissolved in a buffer containing 20 mM sodium phosphate and 150 mM NaCl at pH 6.0 for inhibition assay. First, 90 μL of VraRC [final concentration of 36 μM in buffer (20 mM sodium phosphate and 150 mM NaCl at pH 6.0)] was added to the wells of an ELISA plate. Subsequently, 1 μL of serially diluted inhibitors was mixed with 9 μL of 6-FAM-labeled DNA (final concentration = 10 nM). The inhibitor-DNA solution was further added into the wells of the ELISA plate and incubated for 10 min at 25 °C. Furthermore, the reactions were measured three times using a Synergy H1MF plate reader (BioTek Instruments, Inc.) with an excitation wavelength of 485 nm and an emission wavelength of 535 nm. The inhibition % was derived according to the following equation
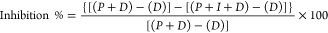
where (*D*) represents the polarization intensity of DNA alone, (*P* + *D*) represents the polarization intensity of VraRC bound to DNA, and (*P* + *I* + *D*) represents the polarization intensity of VraRC mixed with the inhibitor and then incubated with DNA.

### Localized Surface Plasmon Resonance

The binding affinity of the inhibitor to VraRC was evaluated by using an OpenSPR instrument (Nicoya Lifesciences Inc.). To prepare the VraRC protein solution, a Tris-T Buffer (50 mM Tris–HCl pH 7.4, 150 mM NaCl, 0.005% Tween 20) was used. VraRC protein at a concentration of 4.6 μM was immobilized on an NTA sensor chip and then exposed to the inhibitors in the fluid phase. The analyte solutions of the inhibitors were created using Tris-T buffer with 0.5% DMSO and 2% BSA at varying concentrations for detection. Before each experiment, the chip was regenerated by using a 10 mM glycine-HCl buffer at pH 2.2. Finally, the data were fitted to a 1:1 binding model using Trace Drawer software to determine the KD value.

### NMR Spectroscopy and Compound Titrations

The NMR sample of VraRC was prepared at a concentration of 0.3 mM in a buffer containing 20 mM Tris–HCl, 100 mM NaCl, and 1 mM NaN_3_ at pH 8.0. The protein solution was loaded into a Shigemi NMR tube for NMR experiments. The NMR spectra were recorded at 298 K using Bruker AVANCE III 600 MHz spectrometers equipped with a z-gradient TXI cryoprobe (Bruker, Karlsruhe, Germany). Compound titration experiments were performed by adding increasing amounts of the compound to ^15^N-labeled VraRC at pH 8.0, resulting in different protein/compound molar ratios. A series of 2D-^1^H-^15^N heteronuclear single quantum coherence (HSQC) spectra were acquired to monitor the changes in the protein upon compound binding. The VraRC protein titrated with compounds was used to probe the binding site and interactions. All acquired NMR spectra were processed using Bruker TopSpin 4.0 or NMRPipe4^30^ and analyzed using NMRViewJ8.0a.22.5.^31^ The information on backbone NMR chemical shifts of VraRC was retrieved from the Biological Magnetic Resonance Data Bank (accession no.: 51095). The observation of chemical-shift changes in ^1^H,^15^N HSQC of ^15^N-enriched VraRC upon **TST1N-224** titrating was used to confirm interactions and to determine the inhibitor binding site. The weighted chemical shift perturbations (CSPs) for backbone ^15^N and ^1^H_N_ resonances were calculated with the equation Δδ = [((Δδ_HN_)2 + (Δδ_N_/5)^2^)/2]^0.5^.^32,33^

### Molecular Modeling of VraRC-TST1N-224 Complex

We utilized molecular modeling techniques to generate the complex structure of VraRC-**TST1N-224**. The precise binding site for flexible docking of the protein–ligand complex was determined by analyzing residues of VraRC that experienced perturbations during NMR titration with **TST1N-224**. The centroid (*X*, *Y*, Z = 20.547200–6.012750–14.176600) of perturbed residues (M147, E152, T175, K177, T181, S184, I186, L187, K189, L190, Q193, D194, and T196) was employed to define the active site for inhibitor docking. During protein–ligand flexible docking, the GOLD docking program (CCDC, version 5.1) with the GoldScore scoring function was employed. The side chains of binding site residues were allowed to adopt different rotamers to account for the flexibility in the docking analysis. Prior to docking, **TST1N-224** underwent construction and energy minimization. Specific docking parameters, including a set number of operations and a population size (1,600,000 and 1000, respectively), were adjusted, while default settings were retained for other parameters. This approach enabled us to determine the most likely orientation and position of **TST1N-224** within the binding site based on favorable considerations of free energy.

### Determination of Minimum Inhibitory Concentration and Fractioned Inhibitory Concentration Index

The susceptibilities of *S. aureus* (SA), MRSA, and VISA to antibiotics and **TST1N-224** were assessed by determining the minimum inhibitory concentration (MIC), following the guidelines of the National Committee for Clinical Laboratory Standards (CLSI M100 and M07). The experiments employed the broth microdilution method in Mueller–Hinton broth (MHB). During the experiments, each well of 96-well microtiter plates received approximately 100 μL of the inoculum (5 × 10^4^ cfu, final bacterial count) in MHB, and then 100 μL of the test compounds (with interested concentration) was added. The inoculated plates were incubated at 37 °C for 24 h. The MIC was defined as the lowest concentration at which no bacterial growth was observed upon microscopic examination. Furthermore, the 2-dimensional microbroth checkerboard method was employed to examine the in vitro effects of various combinations of tested compounds against VISA.^34^ The tested compounds included **TST1N-224** in combination with either vancomycin or methicillin. In each well of the microtiter plate, an inoculum of 5 × 1 × 10^4^ cfu/ml was incubated at 37 °C for 24 h. The MIC of each tested compound when used alone or in combination represented the lowest dilution at which bacterial growth was completely inhibited. To quantitatively evaluate the interaction of the drugs in combination, the fractional inhibitory concentration (FIC) index (FICI) was calculated for each compound combination. The FICI is determined by summing the FIC of two compounds, where the FIC of each compound is calculated by dividing the MIC when used in combination with the MIC of that compound alone. The FICI results were then interpreted as synergistic (≤0.5), additive (>0.5 to ≤1), or indifferent (>1).^34^

### Adjuvant Experiments

The experiment involves serial dilution based on the MIC of the tested compounds. The experiments were carried out in a flat-bottomed, transparent 96-well plate as follows. First, 50 μL of vancomycin was added to the plate followed by the addition of 100 μL of diluted VISA (5 × 10^4^ cfu/ml, final bacterial count). After that, 50 μL of inhibitors of interest concentrations and MHB (as a control) were added into the wells at 0, 2, 4, 6, and 8 h and incubated at 37 °C for 24 h. Subsequently, OD_660_ was measured to monitor the growth of bacteria. Additionally, the time growth curve assays were performed by the same procedures mentioned above with the measurements of OD_660_ every 2 h.

### Evaluation of Cell Viability

In a 24-well plate, OECM-1 oral cancer cells were cultured using a Gibco RPMI-1640 medium supplemented with 10% FBS and antibiotics. Subsequently, the cells were subjected to varying concentrations of **TST1N-224** (0, 50, and 100 μM) and incubated for 0, 2, and 4 days. Following the incubation period, the supernatant was aspirated, and the adherent cells were washed twice with PBS. Staining was performed using a 0.23% crystal violet solution (Sigma) for 10 min, followed by two additional washes. The plate was air-dried, and cell dissolution was carried out by using a 1% SDS solution. Microplate reader quantification at 562 nm was employed to determine the cell viability. The values obtained from the mock control were set as 100% to facilitate the calculation of the relative cell viability.

## Results

### Receptor–Ligand Pharmacophore Generation

**Figure 1:**
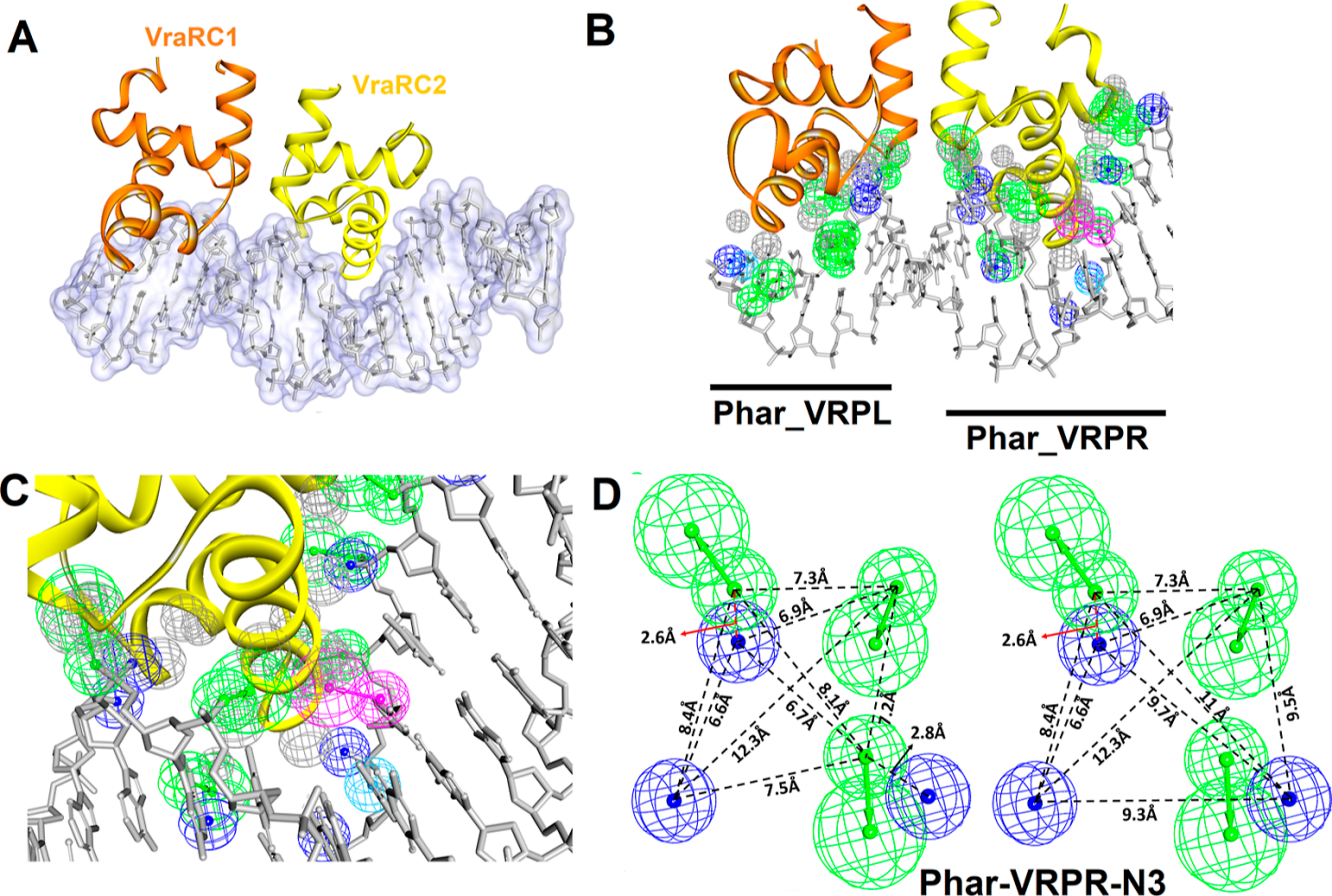
Receptor–ligand pharmacophore generation based on the structure of VraRC-DNA complex. (A) Construction of pharmacophore models using the complex structure of VraRC-DNA (PDB ID: 7VE5). The protein structure is represented as ribbons, and the DNA molecule is displayed as sticks. (B) Depiction of generated pharmacophore features in conjunction with the VraRC-DNA complex structure. Pharmacophore features are color-coded: green for hydrogen-bond acceptor, magenta for hydrogen-bond donor, and deep-blue for negatively charged features. (C) Detailed view of **Phar-VRPR**. (D) Features at specific distances corresponding to the pharmacophore model, **Phar-VRPR-N3**.

To efficiently identify potent inhibitors against VraR, it is crucial to consider the functionally essential features that play key roles in the interactions between VraR and its target DNA. Pharmacophore modeling is a powerful technique for identifying and characterizing crucial elements within a ligand, ensuring precise and effective binding to a receptor.^35,36^ Receptor–ligand pharmacophore generation involves the translation of protein properties into corresponding ligand features.^37^ This method can be utilized to investigate the essential functional features in DNA and target protein interactions. Presently, the X-ray structure of phosphorylated VraR has been determined. However, the complex structure of phosphorylated VraR-DNA has not been solved, hindering the use of the VraR-DNA complex structure for pharmacophore modeling and subsequent pharmacophore-based inhibitor screening. Recently, the structure of the VraRC-DNA complex was determined and is available for analysis. Therefore, we used the complex structure of VraRC-DNA (PDB ID: 7VE5) (Figure 1A) to construct the pharmacophore model using receptor–ligand pharmacophore generation. In this process, VraRC was employed as the receptor, while the DNA structure was used as the ligand to construct the pharmacophore model. Subsequently, two distinct clusters of pharmacophore features were successfully generated and designated as **Phar-VRPL** and **Phar-VRPR** (Figure 1B). **Phar-VRPL** comprises 5 hydrogen-bond acceptors (depicted as green spheres), 3 negative-charged features (represented by blue spheres), and 1 hydrophobic feature (illustrated as a cyan sphere) (Figure 1B). **Phar-VRPR** encompasses 7 hydrogen-bond acceptors, 1 hydrogen-bond donor (depicted as magenta spheres), 1 hydrophobic feature, and 7 negative-charged features (Figure 1B). These pharmacophore models offer a comprehensive representation of the essential features necessary for DNA binding and interaction with VraRC and are useful for identifying and designing potential inhibitors.

### Pharmacophore-Based Inhibitor Screening

**Figure 2:**
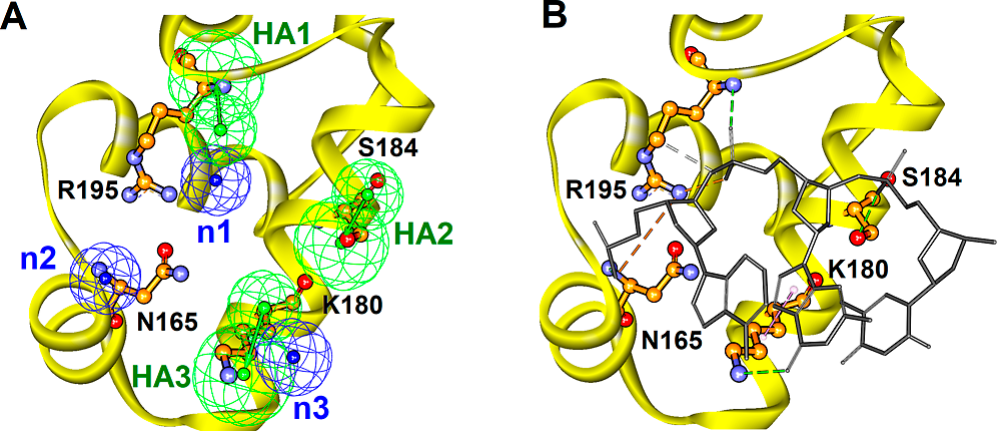
Schematic representations of pharmacophore model, Phar-VRPR-N3. (A) Amplified view of pharmacophore model, **Phar-VRPR-N3**. The protein is presented as ribbon, and the interactive residues are shown as sticks (orange) and labeled. Pharmacophore features are color-coded: green for hydrogen-bond acceptor, magenta for hydrogen-bond donor, and deep-blue for negatively charged features. (B) Molecular interactions of the functional residues of VraRC binding to DNA. The DNA molecule is shown as thin sticks (gray).

**Figure 3:**
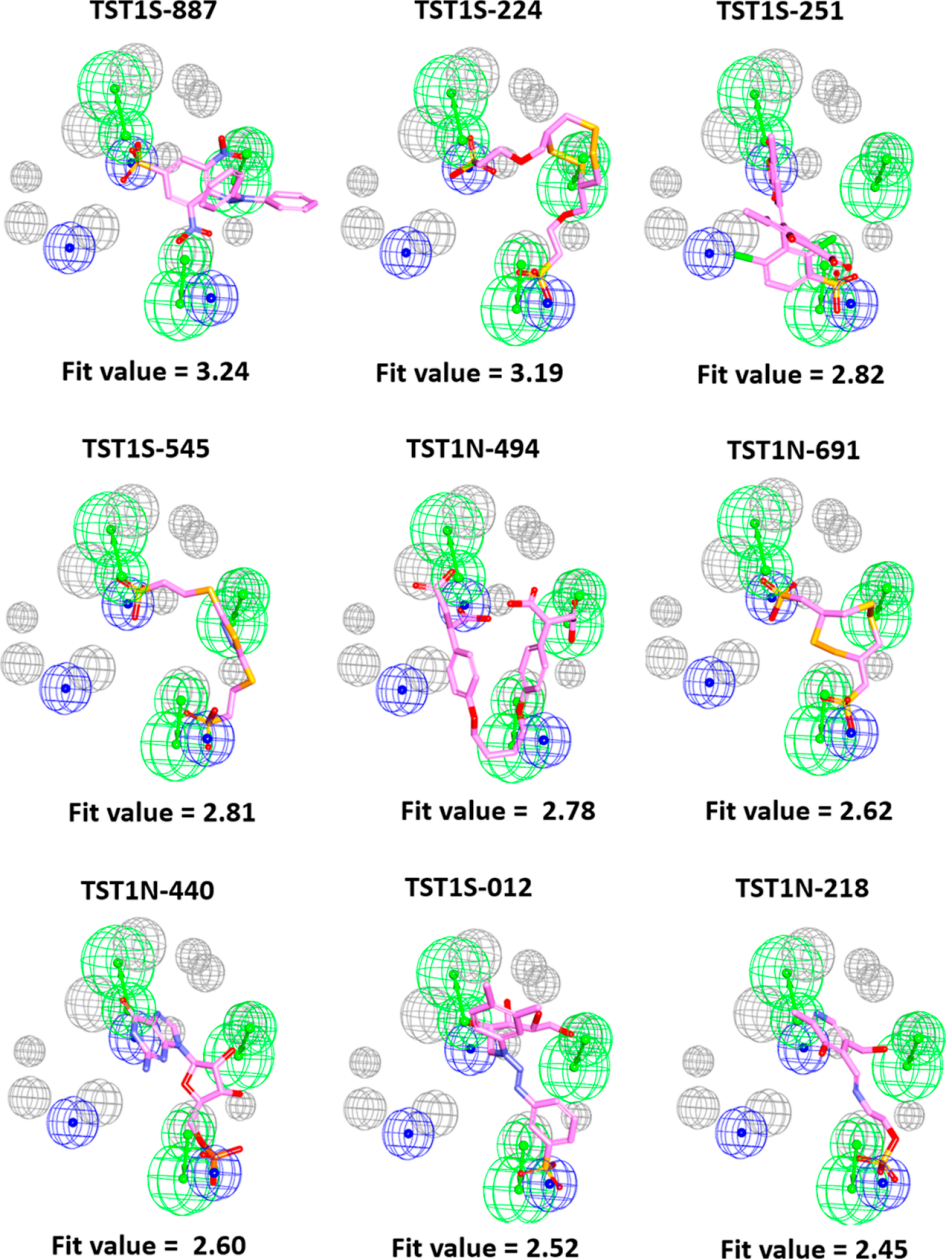
Pharmacophore-based inhibitor screening. Illustration of the results from ligand pharmacophore (**Phar-VRPR-N3**) mapping for hits screened from the IBS database. The top 9 ranked hits are aligned with the pharmacophore model, **Phar-VRPR-N3**. Pharmacophore features are color-coded: hydrogen-bond acceptor in green and negative charge in deep-blue.

Efficiently screening inhibitors through pharmacophore modeling necessitates the careful selection of a pharmacophore scaffold for ligand-pharmacophore mapping. Consequently, we undertook a comprehensive examination of the pharmacophore properties associated with **Phar-VRPL** and **Phar-VRPR**. Our analysis unveiled that a DNA bioactive scaffold, which interacts with residues N165, K180, S184, and R195 (Figure 2A,B), can be represented by three negatively charged features (**n1**, **n2**, and **n3**) and 3 hydrogen-bond acceptors (**HA1**, **HA2**, and **HA3**) (Figure 2). The features of **Phar-VRPR** were further consolidated and organized into a pharmacophore scaffold called **Phar-VRPR-N3** (Figures 1D and 2). This scaffold was then utilized to screen a compound library consisting of 68,000 molecules obtained from the IBS database. The ligand-pharmacophore mapping process was carried out to screen and align these compounds onto the **Phar-VRPR-N3** scaffold. In the process of ligand-pharmacophore mapping, the 3D coordinates of the ligands are aligned with the pharmacophore features of **Phar-VRPR-N3**. This alignment allows for assessment of the fit between the ligand and the pharmacophore. Fit values are assigned to indicate the quality of the match between the ligand and the pharmacophore, with higher scores indicating a stronger and more favorable fit. Consequently, the top 9 ranked hits from the screening process were chosen as potential candidates (Figure 3). The fit values follow the following hierarchy: **TST1S-887** > **TST1N-224** > **TST1S-251** > **TST1S-545** > **TST1N-494** > **TST1N-691** > **TST1N-440** > **TST1S-012** > **TST1N-218**. The detailed chemical structures of these identified candidates are displayed in Figure S1.

### Disruptive Ability of Inhibitors to the Formation of VraRC-DNA Complex

**Figure 4:**
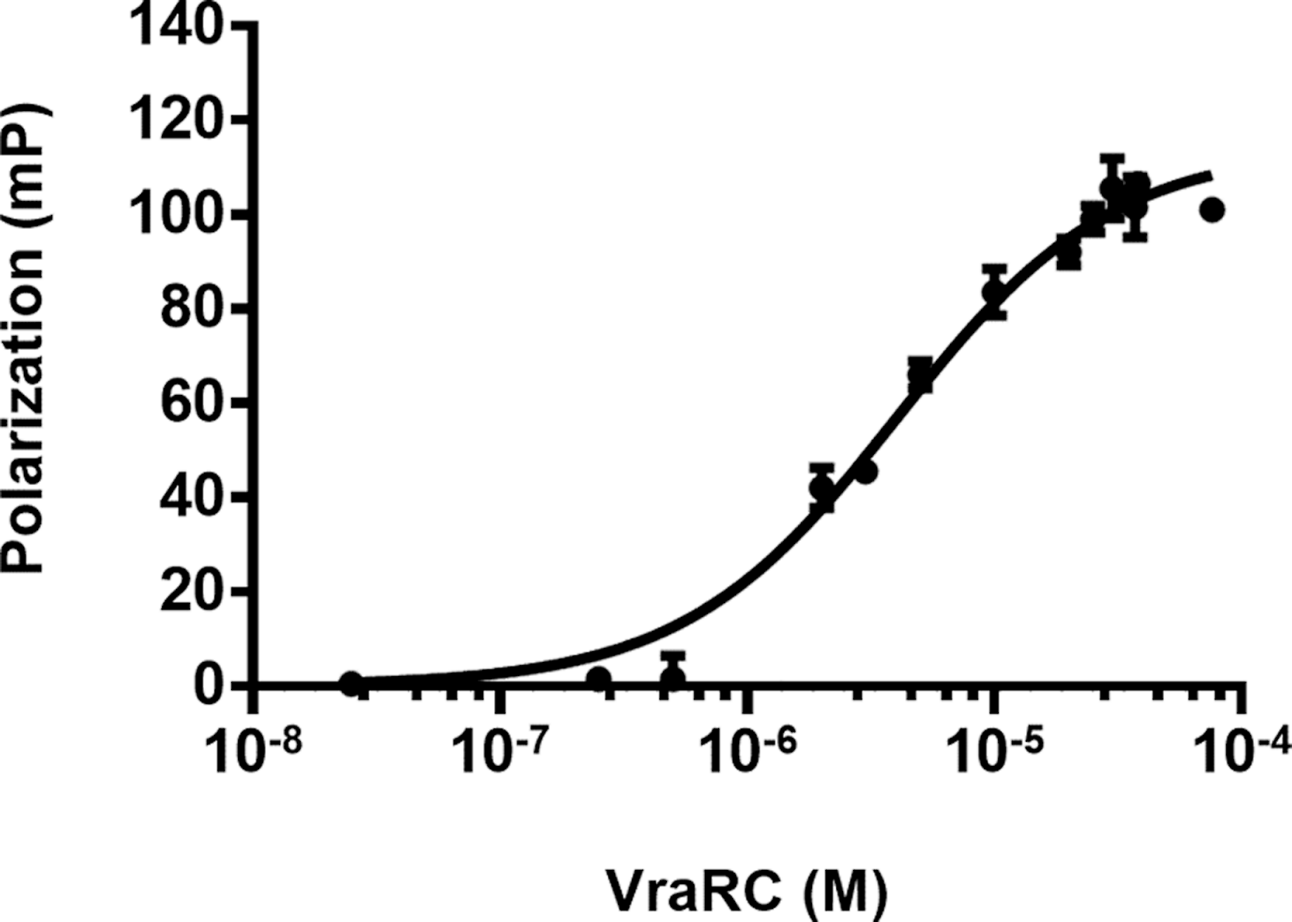
DNA binding property of VraRC. The DNA binding ability of VraRC is observed by FP experiments as a function of protein concentration. The determined KD value is 4.1 ± 0.37 μM.

**Figure 5:**
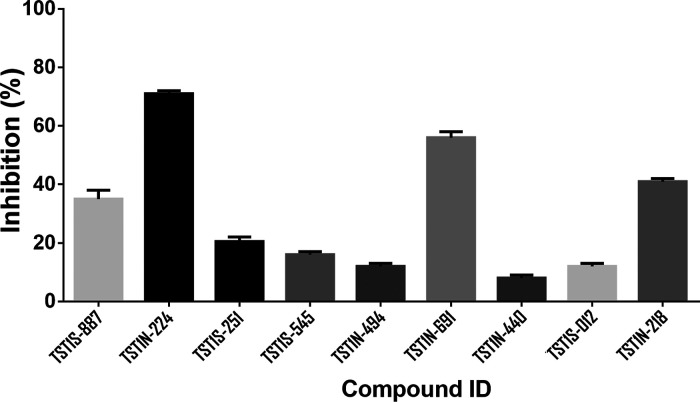
Inhibitory potency of top 9 ranked hits against the complex formation of VraRC-DNA. Evaluation of the inhibitory capacity of the top 9 ranked hits against the formation of the VraRC-DNA complex at 100 μM concentration is depicted.

**Figure 6:**
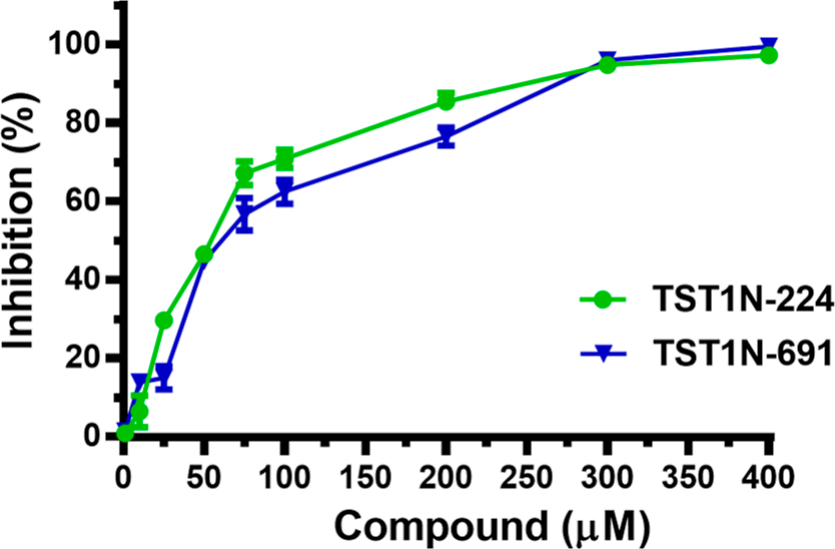
Inhibitory potencies of TST1N-224 and TST1N-691 as a function of compound concentration. The dose-dependent inhibition curves of **TST1N-224** and **TST1N-691** against the formation of VraRC-DNA complex are shown.

To assess compounds’ ability to inhibit VraR binding to DNA, we initially expressed and purified full-length VraR for assays. However, due to strict regulatory controls on BeCl_2_ [required to generate BeF_3_^–^ (a phosphate analogue for proteins phosphorylated on aspartate)] in our country, we cannot experimentally activate VraR. Alternatively, VraRC showed greater stability during protein preparation, facilitating subsequent experiments. Moreover, the availability of the VraRC-DNA complex structure allows for pharmacophore modeling and inhibitor screening focused on the DNA-binding domain, simplifying the identification of inhibitors that disrupt VraR-DNA binding. Therefore, we evaluated the inhibitory activity of identified compounds against VraRC binding to DNA using fluorescence polarization experiments. Prior to these experiments, the interaction of VraRC with DNA was initially characterized to provide a basis for the inhibition assay. The results demonstrated an increase in polarization intensity as the protein concentration of VraRC increased, as depicted in Figure 4. The result indicated that VraRC strongly binds to DNA, with a KD value of 4.1 ± 0.37 μM (Figure 4). Notably, the polarization intensity reached a plateau when the concentration of VraRC is around 36 μM. Therefore, a concentration of 36 μM VraRC was employed for the inhibition assay. Subsequently, the inhibitory abilities of the top 10 ranked hits screened from the ligand-pharmacophore mapping were evaluated at a compound concentration of 100 μM. The results showed that the compounds **TST1N-224** and **TST1N-619** exhibited inhibitions of over 50% (Figure 5). Conversely, **TST1N-218** displayed an approximately 40% inhibition. On the other hand, **TST1S-887**, **TST1N-251**, **TST1S-545**, **TST1S-012**, **TST1S-494**, and **TST1S-938** demonstrated lower or no inhibition against the binding of VraRC to DNA (Figure 5). Moreover, compounds that demonstrated inhibitions exceeding 50% were subjected to further inhibitory experiments, varying the compound concentrations, to determine their IC_50_. Regarding the inhibition of binding of VraRC to DNA, **TST1N-224**, and **TST1N-691** displayed dose-dependent inhibitions, as shown in Figure 6. The IC_50_ values for **TST1N-224** and **TST1N-691** were determined to be 60.2 ± 4.0 and 75.2 ± 6.2 μM, respectively.

### Binding Affinity of TST1N-224 toward VraRC

**Figure 7:**
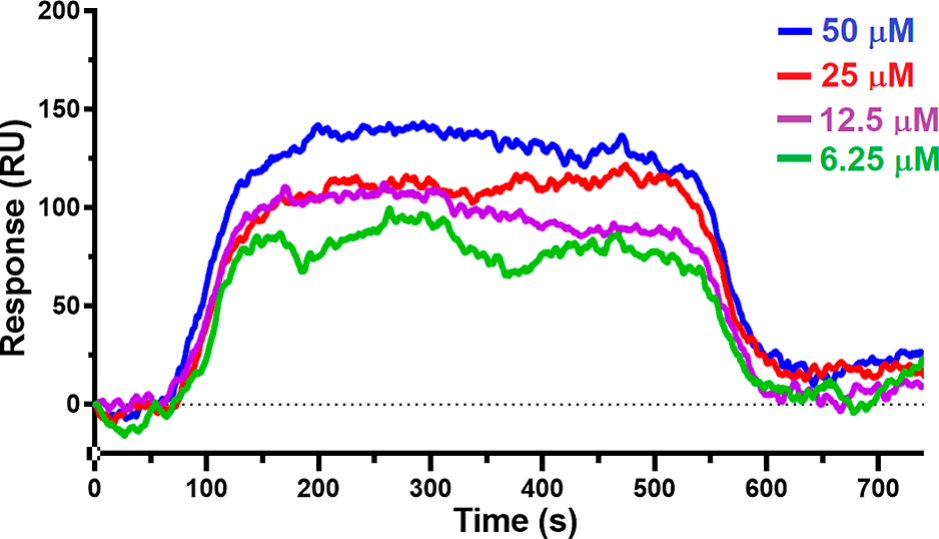
LSPR sensorgrams of TST1N-224 binding to VraRC. The binding affinity of **TST1N-224** (KD = 23.4 ± 1.2 μM) to VraRC.

In our pharmacophore-based inhibitor screening, we successfully identified **TST1N-166** and **TST1N-224** as potent inhibitors against VraRC. To confirm the binding between inhibitors and VraRC, LSPR experiments were performed. During the experiments, **TST1N-224** was tested at concentrations of 6.25, 12.5, 25, and 50 μM against VraRC. The sensorgrams revealed a fast association and fast dissociation binding pattern of **TST1N-224** with VraRC, resulting in a KD value of 23.4 ± 1.2 μM (Figure 7). Similarly, the binding of **TST1N-691** to VraRC was investigated at concentrations of 6.25, 12.5, 25, and 50 μM. However, the sensorgrams showed a very minor and weak binding signal, compared to that of the buffer blank (data not shown). The association signal of **TST1N-691** to VraRC was observed not significantly increasing even if the compound concentration increased to 100–200 μM (data not shown).

### Exploring Inhibitor Binding Site by NMR Spectroscopy

**Figure 8:**
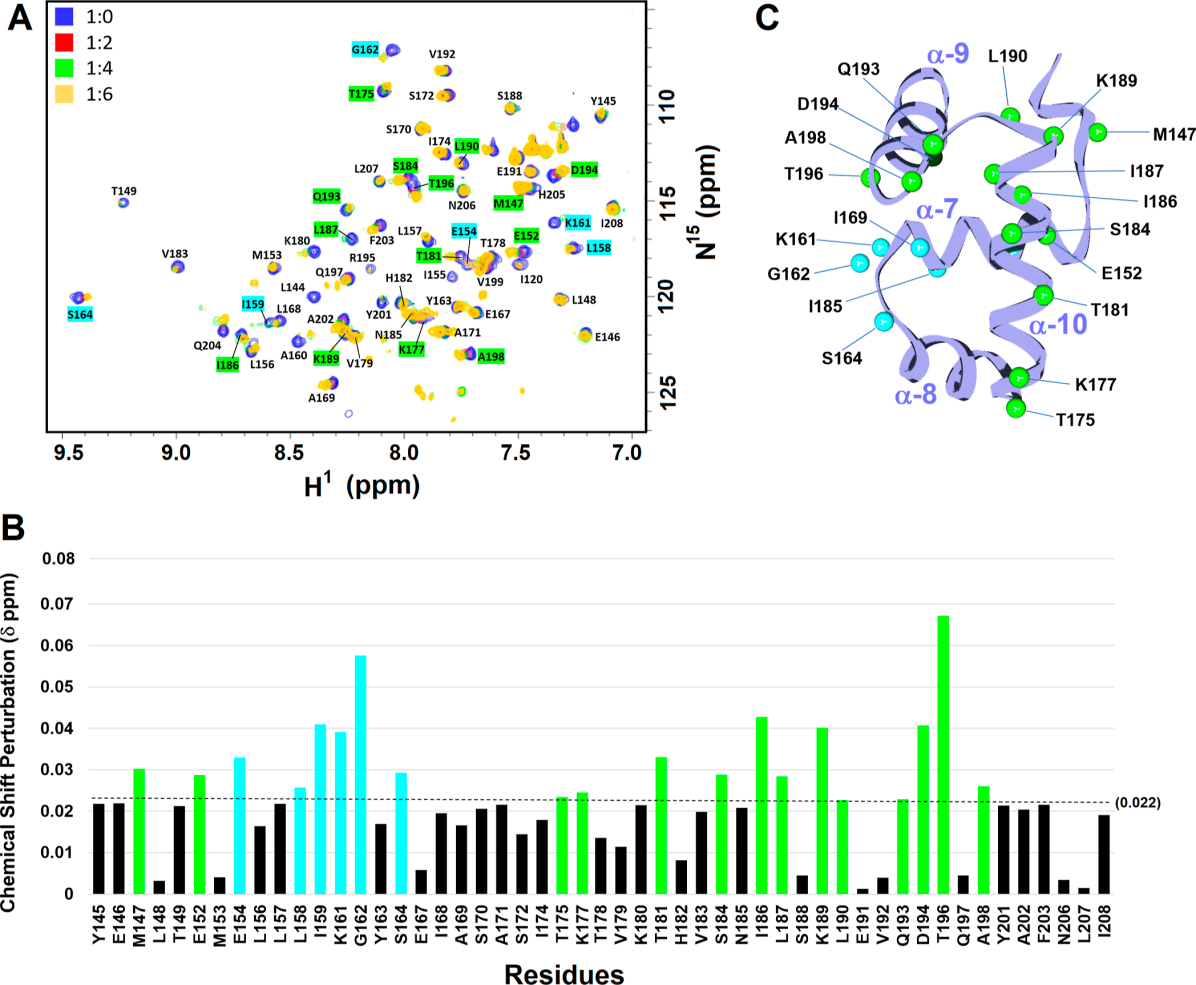
Determination of binding site of TST1N-224 toward VraRC by NMR titrations. (A) Acquired 2D ^1^H–^15^N HSQC spectra of VraRC with the addition of distinct concentrations of **TST1N-224** are overlapped and shown. The molar ratios of VraRC to **TST1N-224** were set to 1:0, 1:2, 1:4, and 1:6. The perturbed residues of VraRC upon **TST1N-224** binding are shown and labeled (highlighted with green and cyan colors). (B) CSP values for backbone amide resonances of VraRC on titration with **TST1N-224** (1:4). Green and cyan bars indicate residues with CSP values more than the average (0.022). (C) Cartoon structure of VraRC showing chemical shift-perturbed residues upon titration of **TST1N-224** highlighted in green and cyan. The protein structure of VraRC is presented as ribbon, and the Cα atoms of each perturbed residues are shown as spheres.

To explore the inhibitor binding site, we characterized the molecular interactions of **TST1N-224** with VraRC by NMR-HSQC. First, the chemical structure and purity of **TST1N-224** were verified and confirmed by high-resolution electrospray ionization mass spectrometry, high-performance liquid chromatography, and nuclear magnetic resonance spectroscopy (Figures S2–S4). Subsequently, the titrations of **TST1N-224** toward VraRC were carried out at the molar ratios of protein to inhibitor = 1:0, 1:2, 1:4, and 1:6 to acquire the HSQC spectra, respectively. The results showed that with the additions of **TST1N-224**, the HQSC spectra of VraRC all showed CSPs (Figure 8A). The determined average CSP of residues of VraRC upon **TST1N-224** titration (molar ratio of protein: inhibitor = 1:4) was determined to be 0.022. Therefore, residue with a CPS value ≥ 0.022 is defined to be most perturbed. The most perturbed residues are M147, E152, E154, L158, I159, K161, G162, S164, T175, K177, T181, S184, I186, L187, K189, L190, Q193, D194, T196, and A198 (Figure 8B). These interactive residues of VraRC pointed out the possible binding site of **TST1N-224** (Figure 8C).

### Complex Structure of VraRC-TST1N-224

**Figure 9:**
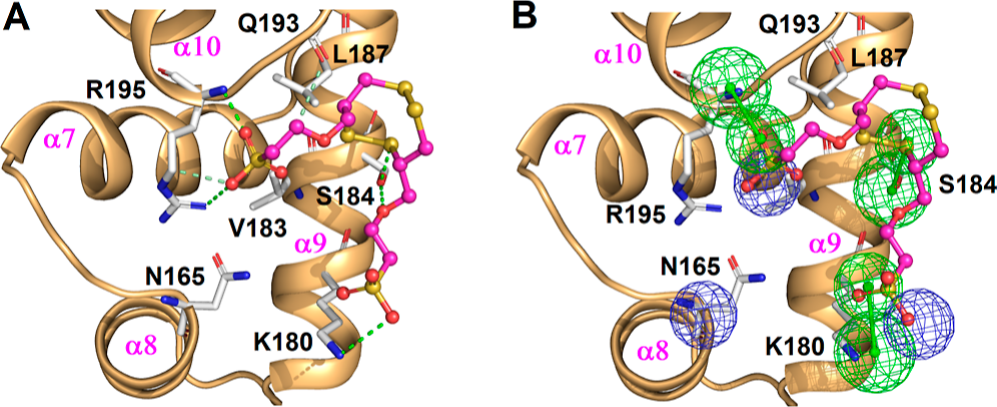
Complex structure of VraRC-TST1N-224. (A) The complex structure of VraRC-**TST1N-224** was built by NMR-based molecular modeling. **TST1N-224** binds between α9 and α10-helix of VraRC. The residues of VraRC interacting with **TST1N-224** are shown as sticks (white) and labeled (black). VraRC is presented as ribbon, and **TST1N-224** is shown as sticks (magenta). (B) The complex structure of VraRC-**TST1N-224** is aligned with the pharmacophore model, **Phar-VRPR-N3**.

To gain a more comprehensive understanding of the atomic-level interactions between VraRC and the potent inhibitor **TST1N-224**, we utilized molecular modeling techniques to construct the complex structure. The residues (M147, E152, T175, K177, T181, S184, I186, L187, K189, L190, Q193, D194, and T196) in VraRC that displayed perturbations during the NMR titration with **TST1N-224** were identified as the binding site for the subsequent protein–ligand flexible docking. During protein–ligand flexible docking, we allowed for the flexibility of side chains in the binding site residues to explore various rotamers. Eventually, we selected the model with the lowest energy, where **TST1N-224** conformed closely to the characteristics of **Phar-VRPR-N3**, as the final complex structure of VraRC-**TST1N-224**. The built complex structure was further analyzed by nonbond interaction analysis (Discovery Studio 2021) to unveil the detailed molecular interactions. The results showed that **TST1N-224** was positioned between the α9- and α10-helixes of VraRC. The specific molecular interactions between **TST1N-224** and VraRC are visualized in Figure 9. Notably, in this binding orientation, the terminal sulfonic groups of **TST1N-224**, which correspond to **n1** and **n3** of **Phar-VRPR-N3**, engaged in charge–charge interactions with residues R195 and K180. Remarkably, residue R195 demonstrated interactions with **TST1N-224** through a carbon–hydrogen bond and an additional hydrogen bond. Additionally, the S1 atom on 1,2,5,6-tetrathiocane and the O7 atom of **TST1N-224** formed hydrogen bonds with the side chain of residue S184. Furthermore, the C22 atom of **TST1N-224** interacted with Q193 through a carbon–hydrogen bond (Figure 9).

### In Vitro Inhibition of TST1N-224 against MRSA and VISA

To test the biological activity of **TST1N-224**, the growth of VISA was observed with the addition of inhibitor to determine the MIC. Meanwhile, the susceptibility of standard strains of SA and MRSA to **TST1N-224** were also tested. The experiments were performed with a final bacterial count of 5 × 10^4^ cfu/ml and followed the guidelines of CLSI (M100 and M07). The results showed that **TST1N-224** can inhibit the growths of SA (MIC > 126 μM), MRSA (MIC > 126 μM), and VISA (MIC = 63 μM) (Table 1). In addition, the MICs of vancomycin complied with the concentrations specified in the CLSI (M100). These results revealed that **TST1N-224** exerted better antibacterial effects on VISA.

**Table 1:** MIC of Antibacterial Drugs (μg/mL)

strains	TST1N-224	methicillin	vancomycin
Staphylococcus aureus (SA)	>64 (126 μM)	0.25	0.5
methicillin-resistant Staphylococcus aureus **(MRSA)**	>64 (126 μM)	0.5	1
vancomycin-intermediate Staphylococcus aureus Z172 (VISA)	32 (63 μM)	64	2

### Synergetic Effects of TST1N-224 Combined with Methicillin or Vancomycin against VISA

The FICI is an experimental method based on the MIC to investigate the synergistic effects of drugs in inhibiting bacteria. By using this, we can explore the biological function of **TST1N-224** in combination with vancomycin and/or methicillin against VISA. The results showed that the combination of **TST1N-224** and vancomycin led to a FICI > 1.0, indicating no synergistic effect on the growth of bacteria (Figure S5). In contrast, the growth of VISA was significantly inhibited when treated with the combination of **TST1N-224** and methicillin (FICI = 0.675) (Figure S6 and Table 2). This indicates an additive effect on bacterial growth, implying that **TST1N-224** has a better synergistic effect when combined with β-lactam antibiotics such as methicillin.

**Table 2:** FICI of Methicillin with TST1N-224 against *Staphylococcus aureus* subsp. aureus Z172 (VISA)^a^

methicillin (μg/mL)	TST1N-224 (μg/mL)	FIC_A_ (methicillin)	FIC_B_ (TST1N-224)	FICI	effect
64	4 (7.9 μM)	0.5	0.125	0.675	additive effects

a MIC of methicillin: 128 μg/mL; MIC of **TST1N-224**: 32 μg/mL. VISA culture: 5 × 10^4^ cfu/ml. FICI = FIC_A_ + FIC_B_. FICI index of: < 0.5, synergism; > 0.5–1, additive effects; >1 to <2 indifference; ≥ 2 antagonism.

### Cytotoxicity of TST1N-224

**Figure 10:**
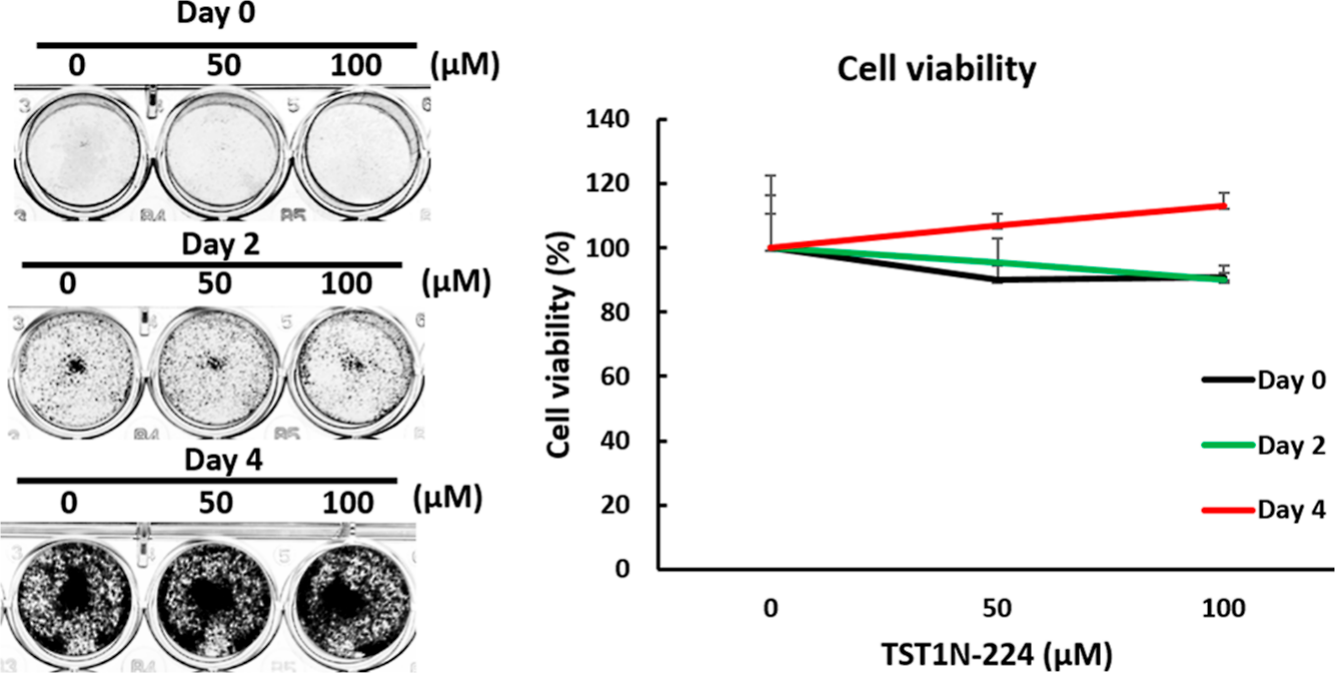
Cell viability of oral cancer cell, OECM-1, under the treatments of TST1N-224.

To reveal the possibility of **TST1N-224** as a drug for the treatment of *Staphylococcus* species, its safety profile was investigated. Oral cancer cell line OECM-1 was treated with 0, 50, or 100 μM of **TST1N-224** for 0, 2, and 4 days. The results showed that cell viability of the condition treated with **TST1N-224** (100 μM) showed no significant change, compared to that of the mock control (Figure 10). This result indicated that **TST1N-224** has no apparent cytotoxicity.

## Discussion

Modern antimicrobial treatment faces one of its most substantial hurdles due to the frequent connection between MRSA and healthcare-associated infections, coupled with MRSA’s capacity to adapt and resist antimicrobial agents. Nowadays, vancomycin stands as the foremost option for combating MRSA infections. However, the excessive utilization of vancomycin has given rise to the emergence of resistant strains, specifically VISA and VRSA. The MIC for VISA typically falls within the range of 4–8 μg/mL, whereas for VRSA, it exceeds 16 μg/mL.^38^ This escalating threat of drug-resistant bacteria poses a significant global public health concern. Addressing bacterial pathogenesis requires the development of innovative bactericidal agents. One promising strategy is to control the pathogenic behavior rather than solely focus on bacterial eradication. TCSs play a crucial role in governing various bacterial behaviors like biofilm formation, virulence, and antibiotic resistance.^39−46^ Therefore, targeting bacterial TCS through inhibitors that deactivate bacterial signaling provides a novel strategy against antibiotic resistance.^47−49^ Numerous efforts have been invested in identifying HK inhibitors, including extensive screening of chemical libraries and structure–activity relationship (SAR) programs for lead compounds.^48,50^ However, small-molecule HK inhibitors often exhibit poor bioavailability owing to their highly hydrophobic properties.^47^ Additionally, some inhibitors lack selectivity^48,51^ and have been reported to influence protein aggregation rather than inhibiting HK.^52^ A distinct advantage lies in targeting the RR instead of HK. Inhibiting RR directly interferes with bacterial gene expression, impacting bacterial behavior.^46^ This alternative approach offers several benefits over conventional strategies and holds promise for the development of antimicrobial agents. Notably, *S. aureus* features a key two-component system with the response regulator VraR and histidine kinase VraS, playing a pivotal role in vancomycin resistance. Genetic modifications in VraS or VraR have been shown to restrict antibiotic resistance to β-lactam and glycopeptide antibiotics in diverse *S. aureus*.^53−55^ Hence, compounds aimed at inhibiting VraS and/or VraR, thereby disrupting the signal transduction pathway linked to antibiotic resistance, hold significant promise in reinstating the susceptibility of VISA or VRSA to vancomycin. In this study, we utilized the VraRC-DNA complex structure as the foundation for a comprehensive exploration to identify a potent inhibitor. We uncovered a distinct binding site and inhibitory mechanism targeting the RR, VraRC, of VISA. Our investigative approach involved the application of pharmacophore modeling, structure-based molecular docking, and thorough biophysical and biochemical examinations, offering a representative analysis.

CADD, which includes molecular docking and pharmacophore modeling, serves as a cost-effective tool for efficiently screening compounds with specific biological functions.^56,57^ Pharmacophore modeling, particularly receptor–ligand pharmacophore generation (structure-based pharmacophores or SBPs), translates protein properties into corresponding ligand features, enabling the development of inhibitors with precise characteristics for effective binding to the target protein.^35,36^ Therefore, structural information from the VraRC-DNA complex is crucial for screening potential inhibitors. In this context, we used the complex structure of the VraRC-DNA (PDB ID: 7VE5) to construct pharmacophore models. Using VraRC as the receptor and DNA as the ligand, we built the pharmacophore models comprising distinct pharmacophore features, **Phar-VRPL** and **Phar-VRPR**, which include hydrogen-bond acceptors, negative-charged features, hydrophobic features, and hydrogen-bond donors (Figure 1B). These features provide a comprehensive representation of the essential attributes necessary for ligand binding and interaction with the VraRC-DNA complex, which is useful for the identification and design of potential inhibitors. Additionally, a DNA bioactive scaffold, which interacts with specific residues (N165, K180, S184, and R195), can be effectively represented by three negatively charged features (**n1**, **n2**, and **n3**) and three hydrogen-bond acceptors (**HA1**, **HA2**, and **HA3**) (Figure 2). These features formed the basis for creating a pharmacophore scaffold, known as **Phar-VRPR-N3**. Furthermore, the top 9 ranked hits (Figure 3) screened by ligand-pharmacophore (**Phar-VRPR-N3**) mapping were examined and demonstrated to exhibit varying degrees of inhibitory effects (Figure 5). Further dose-dependent inhibition assay demonstrated that **TST1N-224** and **TST1N-691** exhibited inhibitory abilities against VraRC-DNA complex formation by FP assay (IC_50_ = 60.2 ± 4.0 and 75.2 ± 6.2 μM) (Figure 6). These findings affirm the reliability and precision of the pharmacophore model, **Phar-VRPR-N3**, for screening inhibitors against the formation of VraRC-DNA complex formation. Moreover, the compounds identified, **TST1N-224** and **TST1N-691**, which feature sulfate groups at both termini, may mimic the functional phosphate groups of DNA, engaging in electrostatic interactions with VraRC.

Moreover, the LSPR experiments demonstrated that **TST1N-224** and **TST1N-691** exhibited notably different binding profiles when interacting with VraRC. The **TST1N-224** displayed a concentration-dependent binding pattern, resulting in an evident affinity to VraRC, with a KD value of 23.4 ± 1.2 μM (Figure 7). In contrast, **TST1N-691** showed very weak binding signals, even at higher concentrations, indicating a limited or negligible affinity for VraRC (data not shown). The weak binding of **TST1N-691** indicated that this compound may not effectively interfere with the binding of VraRC to its target DNA. Whereas the observed apparently binding affinity of **TST1N-224** revealed that this compound can effectively interact with VraRC. Further optimization and modification of **TST1N-224** could enhance disrupting VraRC-related processes. To harness the full potential of **TST1N-224**, it is imperative to understand its specific interactions with VraRC. Therefore, we further employed NMR titrations to probe the binding site of **TST1N-224** toward VraRC. The observed chemical shift perturbations in the HSQC spectra of VraRC with the addition of **TST1N-224** signify significant interactions between the two entities (Figure 8). The perturbed residues, including M147, E152, T175, K177, T181, S184, I186, L187, K189, L190, Q193, D194, and T196, were mostly located at helixes α9- and α10 of VraRC (Figure 8C). In contrast, residues E154, L158, I159, K161, G162, and S164 situated at the α7-helix and the α7-α8-loop may be perturbed due to conformational change of VraRC induced by **TST1N-224** binding. Thus, the NMR titration experiments provide crucial information about the potential binding site of **TST1N-224**. This together with the inhibitory ability (IC_50_ = 60.2 ± 4.0 μM) indicates that **TST1N-224** targets the C-terminal DBD of VraR, further interfering with its binding to DNA. These findings revealed the detailed molecular interactions between **TST1N-224** and VraRC, with the altered residues likely playing a role in the binding interface. Moreover, to comprehensively understand the atomic interactions between VraRC and **TST1N-224**, we utilized NMR-based molecular modeling to construct the complex structure. Residues (M147, E152, T175, K177, T181, S184, I186, L187, K189, L190, Q193, D194, and T196) of VraRC exhibiting perturbations upon **TST1N-224** titration in NMR were employed to generate a centroid (*X*, *Y*, *Z* = 20.547200–6.012750–14.176600) to define the binding site for subsequent protein–ligand flexible docking. The model with the lowest energy, closely resembling **Phar-VRPR-N3** characteristics, was chosen as the final complex structure (Figure 9). Nonbond interaction analysis revealed that **TST1N-224**, mimicking **Phar-VRPR-N3**, occupied a binding site between the α9- and α10-helices of VraRC (Figure 9). Notable interactions of **TST1N-224** targeting VraRC included charge–charge interactions with R195 and K180, carbon–hydrogen bonding with R195, hydrogen bonding with S184, and carbon–hydrogen bonding with Q193. The observed detailed molecular interactions shed light on the specific binding orientation and key residues involved in the interaction between **TST1N-224** and VraRC. The findings enhance our understanding of how **TST1N-224** disrupts VraRC function, potentially paving the way for the development of a drug targeting VraRC for therapeutic applications.

**Figure 11:**
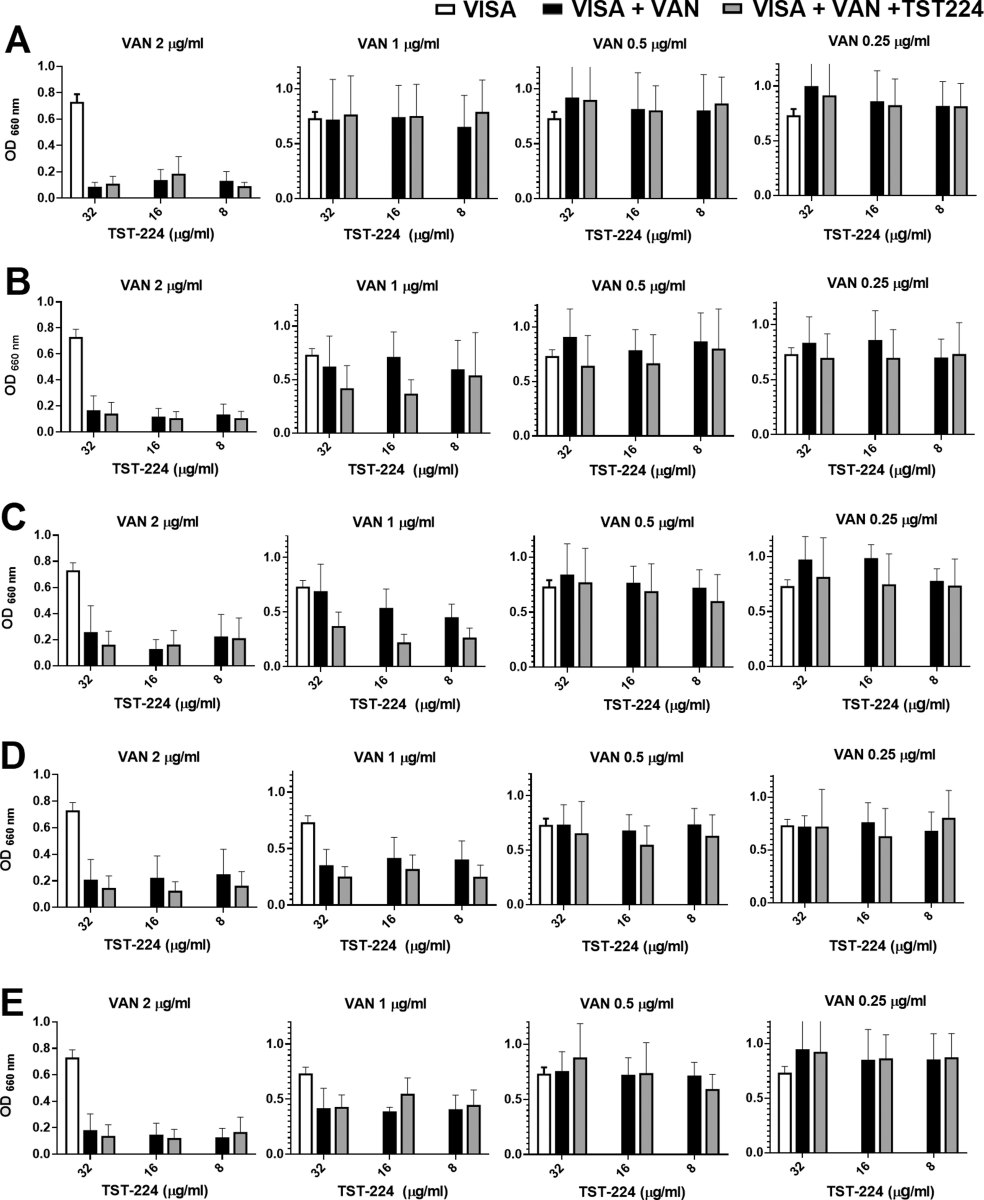
Antibacterial activity of TST1N-224 in combination with vancomycin against VISA. VISA was pretreated with 0.25, 0.5, 1, and 2 μg/mL of vancomycin following by the addition of **TST1N-224** (8 μg/mL (15.75 μM), 16 μg/mL (31.5 μM), and 32 μg/mL (63 μM)) at different time points: (A) 0, (B) 2, (C) 4, (D) 6, and (E) 8 h. The viability of VISA was quantified and indicated by OD_660_. The labels, VISA, VISA + VAN, and VISA + VAN + **TST1N-224** denote VISA without any inhibitor added, VISA with vancomycin treated, and VISA treated with both vancomycin and **TST1N-224**, respectively.

**Figure 12:**
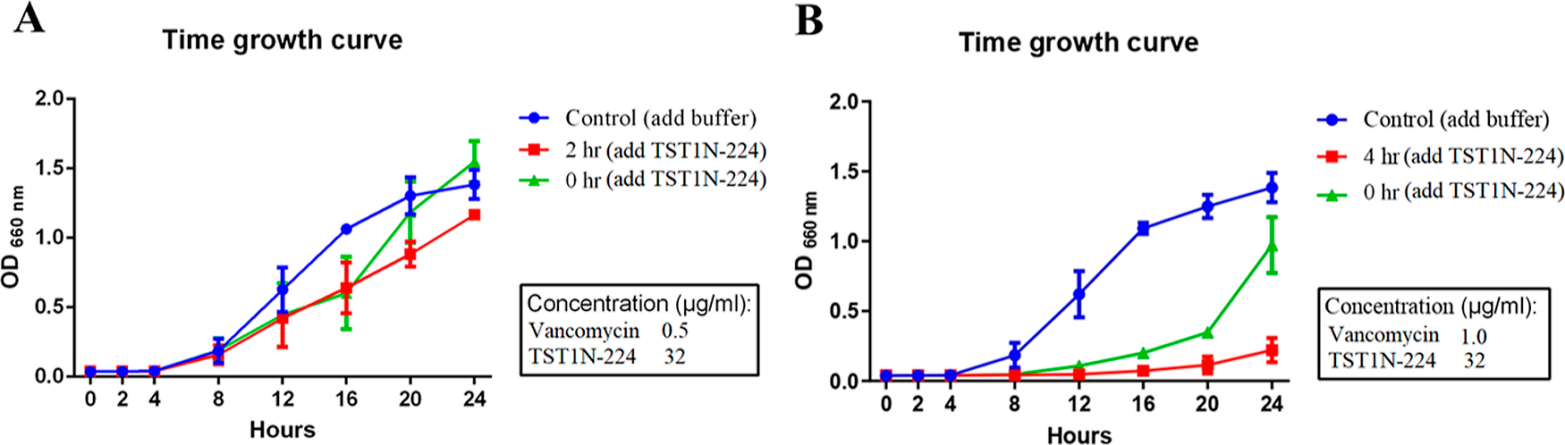
Growth curve of VISA under the treatment of TST1N-224 combined with vancomycin. (A) VISA was pretreated with vancomycin (0.5 μg/mL). After 2 h, **TST1N-224** [32 μg/mL (63 μM)] was added into the culture and incubated at 37 °C to measure OD_660_ to obtain the time course–growth curve. (B) Time course–growth curve of VISA under the treatment of vancomycin (1 μg/mL) followed by the addition of **TST1N-224** [32 μg/mL (63 μM)] at the 4th hour.

TCS inhibitors are anticipated to function either as bactericidal agents or be adapted as adjuvants alongside established antibiotics, targeting colonization, virulence factor expression, and drug resistance.^40,42,58^ To evaluate the impact of **TST1N-224** on the susceptibility of VISA to vancomycin, a microbial viability assay was conducted. The FICI experimental results indicated that the combination of **TST1N-224** and vancomycin exhibits no apparent synergistic impact on inhibiting the growth of VISA. As a result, we hypothesized that the unique chemical structure and characteristics of **TST1N-224** might lead to interactions or binding with vancomycin. Hence, we proceeded to conduct additional adjuvant experiments using **TST1N-224**, focusing on this aspect. The experimental concept is as follows: initially, VISA is treated with vancomycin. Following exposure to vancomycin, there is temporary inhibition of the synthesis of the cell wall precursor, leading to a disruption in the assembly of the cell wall in VISA. Subsequently, at different time intervals, **TST1N-224** is introduced across the bacterial cell membrane to inhibit VraRC, which could further disrupt or abolish the regulation of downstream transcription factors, thereby enhancing the antibacterial effect. Additionally, the time intervals are set to follow the half-life of vancomycin in the human body, which is approximately 4–6 h.^59^ It was assumed that within this specified time frame vancomycin would reach its peak efficacy in breaking down the cell wall. This optimal destruction would facilitate the adjuvant’s easier penetration through the disrupted cell wall and entry into the cell membrane. The experiment used vancomycin dilutions starting from the MIC concentration and followed by adding **TST1N-224** or MHB (control) at 0, 2, 4, 6, and 8 h. The analysis showed minimal or no discernible differences in bacterial growth when **TST1N-224** was added to vancomycin-pretreated VISA (at concentrations of 0.5, 1, 2, and 4 μM) at 0, 6, and 8 h, as compared to the MHB control groups (Figure 11). Conversely, when **TST1N-224** was introduced to vancomycin-pretreated VISA (at concentrations of 2 and 4 μM) at 2 and 4 h, notable enhancements in antibacterial effects were observed (Figure 11B,C). Consequently, an additional time course growth curve assay was conducted, with measurements taken every 2 h at 37 °C for 24 h. The resulting growth curves revealed the ability of **TST1N-224** to enhance the antibacterial effect against VISA when used as an adjuvant. As shown in Figure 12A, VISA was initially treated with vancomycin (1 μM); after 2 h, **TST1N-224** (63 μM) was added to the culture. Bacterial growth was monitored for 24 h, and the OD_660_ difference between the control and experimental groups was 0.2. As shown in Figure 12B, vancomycin (0.69 μM) and **TST1N-224** (63 μM, added at 4 h) were used to treat VISA. After 24 h, the OD_660_ value difference was determined to be 1.05. These results clearly indicate a substantial increase in the antibacterial effect of vancomycin as **TST1N-224** is used as an adjuvant, highlighting the potential efficacy of **TST1N-224**. Interestingly, our BLAST search against the pdbaa database revealed that VraRC of *S. aureus* shares structural and sequence similarity with other RRs in bacteria (Figures S7 and S8). Notably, the conserved region encompasses residues that interact with **TST1N-224,** specifically positions N165, K180, S184, and R195 of VraRC (Figure S8), which are crucial for electrostatic interactions with DNA. The conservation in both structure and sequence at this site suggests that **TST1N-224** may potentially target RRs in other pathogenic bacteria, such as *Mycobacterium tuberculosis* (DosR), *Streptococcus pneumoniae* (S1814), *Enterococcus faecalis* (LiaR), and *Corynebacterium diphtheriae* (Chra) (Figures S7 and S8), and thereby could exhibit broad-spectrum bactericidal activities.

## Conclusions

In conclusion, our investigation utilized a pharmacophore-based methodology, complemented by biochemical and biophysical analyses, to identify inhibitors targeting VISA. The developed pharmacophore model, **Phar-VRPR-N3**, effectively screened 68,000 natural products, culminating in the identification of **TST1N-224** as a potent inhibitor capable of disrupting VraRC-DNA complex formation (IC_50_ = 60.2 ± 4.0 μM). **TST1N-224** exhibited interference with VraRC binding to its cognate DNA through a fast-on-fast-off binding mechanism (KD = 23.4 ± 1.2 μM). The complex structure delineated **TST1N-224’s** preferential interaction with the α9- and α10-helixes of the DNA-binding domain, presenting potential avenues for structure-based lead optimization against other pathogenic bacteria. Characterized by 1,2,5,6-tetrathiocane with positions 3 and 8 modified with ethanesulfonates, **TST1N-224** emerges as a potential foundation for the development of adjuvants or novel antimicrobial agents.

## Data Availability

Structural and computational analysis data sets are publicly accessible and can be found at the following repository: https://github.com/Emersontseng/VraRC.git

## Supporting Information Available

Chemical structures of the identified hits from ligand-pharmacophore mapping; NMR structural data and purity data of **TST1N-224**; FICI of **TST1N-224** and vancomycin against VISA; FICI of **TST1N-224** and methicillin against VISA; multiple sequence alignment among VraRC and RRs of other bacterial species; and structure conservations among VraRC and RRs of other bacterial species (PDF)
