# Supplementary material for: Combatting Antibiotic-Resistant Staphylococcus aureus: Discovery of TST1N-224, a Potent Inhibitor Targeting Response Regulator VraRC, through Pharmacophore-Based Screening and Molecular Characterizations
Source: J Chem Inf Model. 2024 Jul 30;64(15):6132–46. doi: 10.1021/acs.jcim.4c01046 (PMC11323011; doi:10.1021/acs.jcim.4c01046)
Supplement: Supplementary file 1 — ci4c01046_si_001.pdf [file ci4c01046_si_001.pdf]

Supporting information

Combatting Antibiotic-Resistant *Staphylococcus aureus*: Discovery of TST1N-224, a Potent Inhibitor Targeting Response Regulator VraRC, through Pharmacophore-Based Screening and Molecular Characterizations

Ying Chu Hsu[a#], Ching-Hui Liu[b#], Yi-Chen Wu[b#], Shu-Jung Lai[c, d], Chi-Jan Lin[b], and Tien-Sheng Tseng[b*]

[a] Division of Neurology, Department of Internal Medicine, Ditmanson Medical Foundation ChiaYi Christian Hospital, Chiayi, Taiwan.

[b] Institute of Molecular Biology, National Chung Hsing University, Taichung, Taiwan. [c] Graduate Institute of Biomedical Sciences, China Medical University, Taichung, Taiwan.

[d] Research Center for Cancer Biology, China Medical University, Taichung, Taiwan.

[#] Equal contribution

* To whom correspondence should be addressed.

Tel: +886-4-22840485 #270; Fax: +886-4-22874879

Email: emersontseng@dragon.nchu.edu.tw; i90221141ster@gmail.com

Correspondence may also be addressed to emersontseng@dragon.nchu.edu.tw; i90221141ster@gmail.com

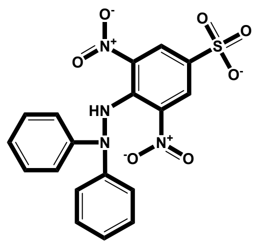

STOCK1S-22887
TST1S-887

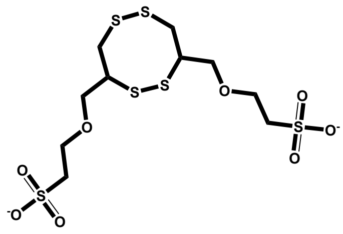

STOCK1N-13224
TST1N-224

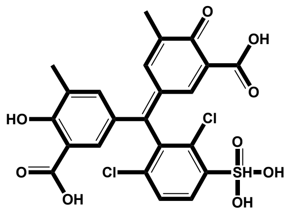

STOCK1S-58251
TST1S251

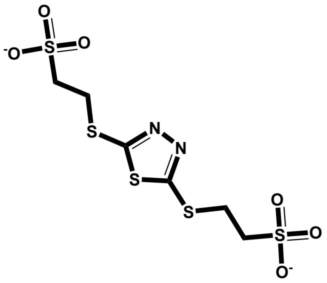

STOCK1S-50545
TST1S-545

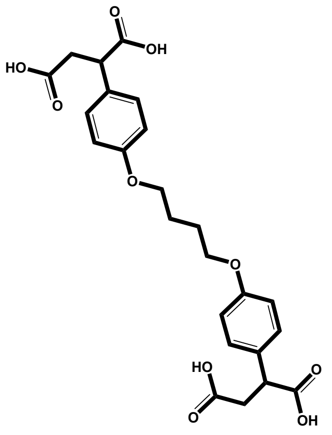

STOCK1N -01494
TST1N-494

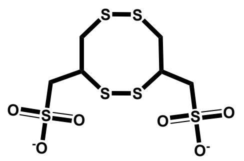

STOCK1N-16691
TST1N-691

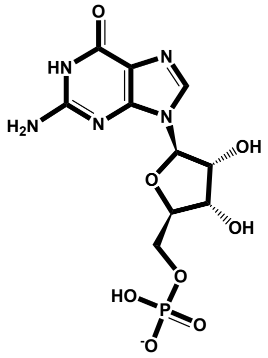

STOCK1N-52440
TST1N-440

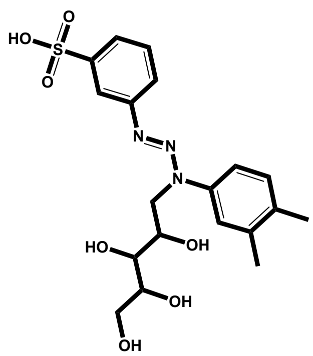

STOCK1S-27012
TST1S-012

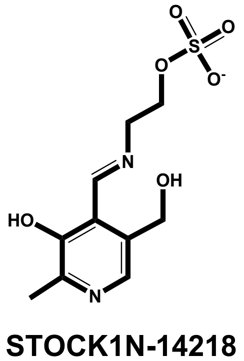

STOCK1N-14218
TST1S-218

Figure S1. The chemical structures of the identified hits from ligand-pharmacophore mapping.

Spectrum

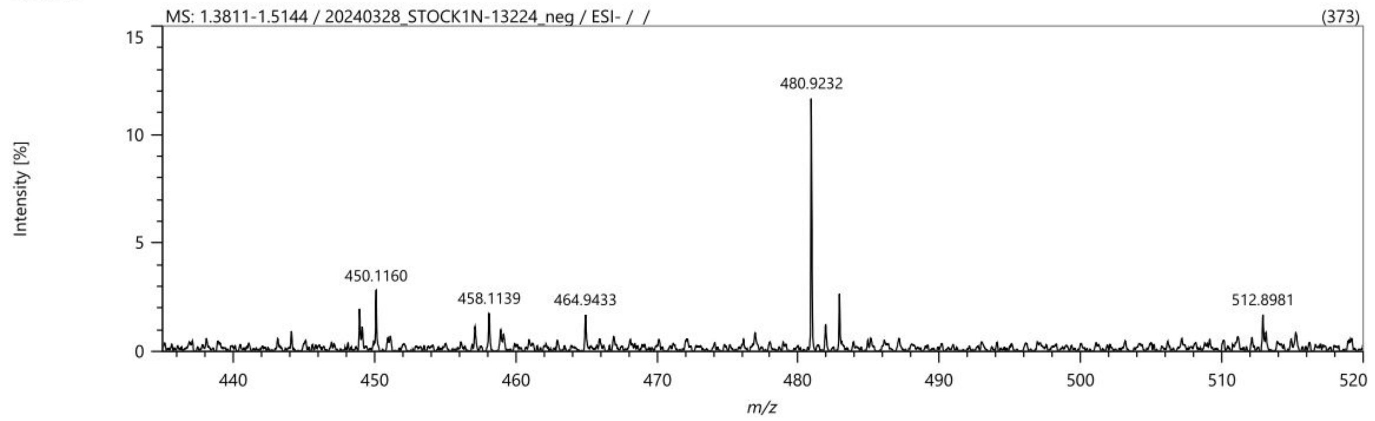

Elemental Composition

Parameters	
Tolerance:	±100.00 ppm
Electron:	Odd/Even
Charge:	-1
DBE:	-99.0 - 999.0

Elements Set 2:

Symbol	C	H	O	S	Na
Min	0	0	8	6	1
Max	400	1000	8	6	1

Results

Mass	Formula	Calculated Mass	Mass Difference [mDa]	Mass Difference [ppm]	DBE
480.92324	C10 H18 O8 Na S6	480.92291	0.33	0.68	1.5

Figure S2. The HR-ESI MS spectrum of TST1N-224. High-resolution electrospray ionization mass spectrometry (HR-ESI MS) was used to verify the molecular weights (MW) of **TST1N-224**. The result indicated that the determined MW for **TST1N-224** was 480.9. The theoretical MWs for **TST1N-224** ($C_{10}H_{18}O_8NaS_6$) is 481. **TST1N-224** was ionized as [M-Na+]- in ESI negative mode, and the high-resolution calibration errors were all within acceptable limits. The high-resolution calibration error was within acceptable limits. Therefore, the MW determined by HR-ESI MS is consistent with the theoretical values. The HR-ESI MS analysis was conducted by the Mass Laboratory at the Institute of Chemistry, Academia Sinica, Taipei, Taiwan. (https://www.chem.sinica.edu.tw/facilities.php?bodyMenu=publicequipments&lang=en).

(A)

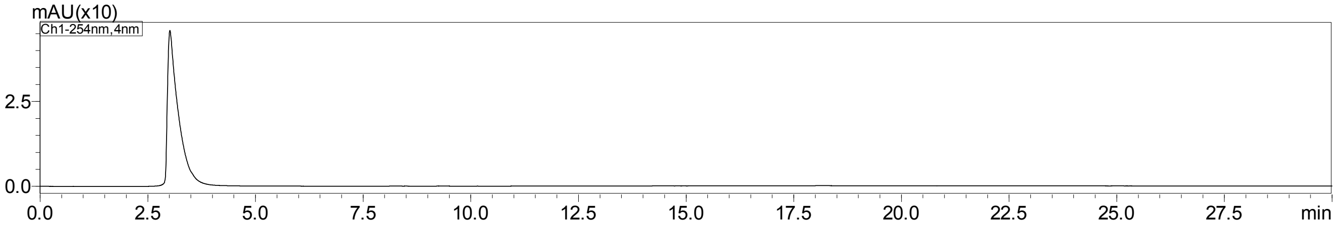

(B)

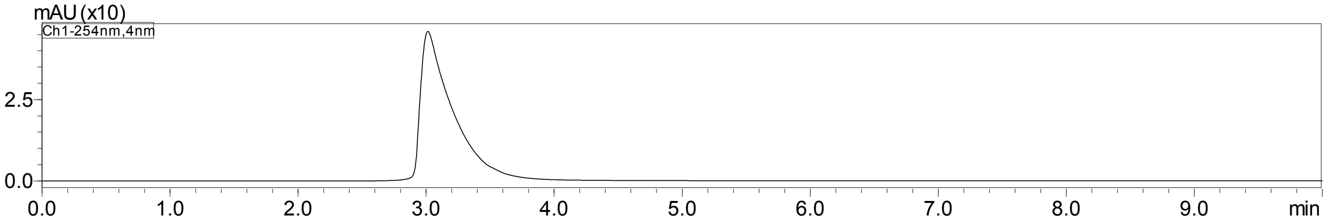

Figure S3. High performance liquid chromatography (HPLC). The purity of **TST1N-224** was verified using HPLC (SHIMADZU LC-2040C - 3D Plus, Shimadzu Corporation). In this HPLC system, methanol was used as the mobile phase, and a Luna 5 µm C18(2) 100 Å LC column (250 x 4.6 mm, Ea, Phenomenex) was used for liquid chromatography. The OD_{254} was employed to detect the absorbance of compound. (**A**) The HPLC profile of **TST1N-224**. The MeOH is used as the mobile phase with a gradient 20~100% (Flow rate = 0.8 ml/min). (**B**) The HPLC profile of **TST1N-224**. The 50% MeOH is used as the mobile phase (Flow rate = 0.8 ml/min). The resulting HPLC profiles, shown in (**A**) and (**B**), confirmed the purity of **TST1N-224**.

(A)

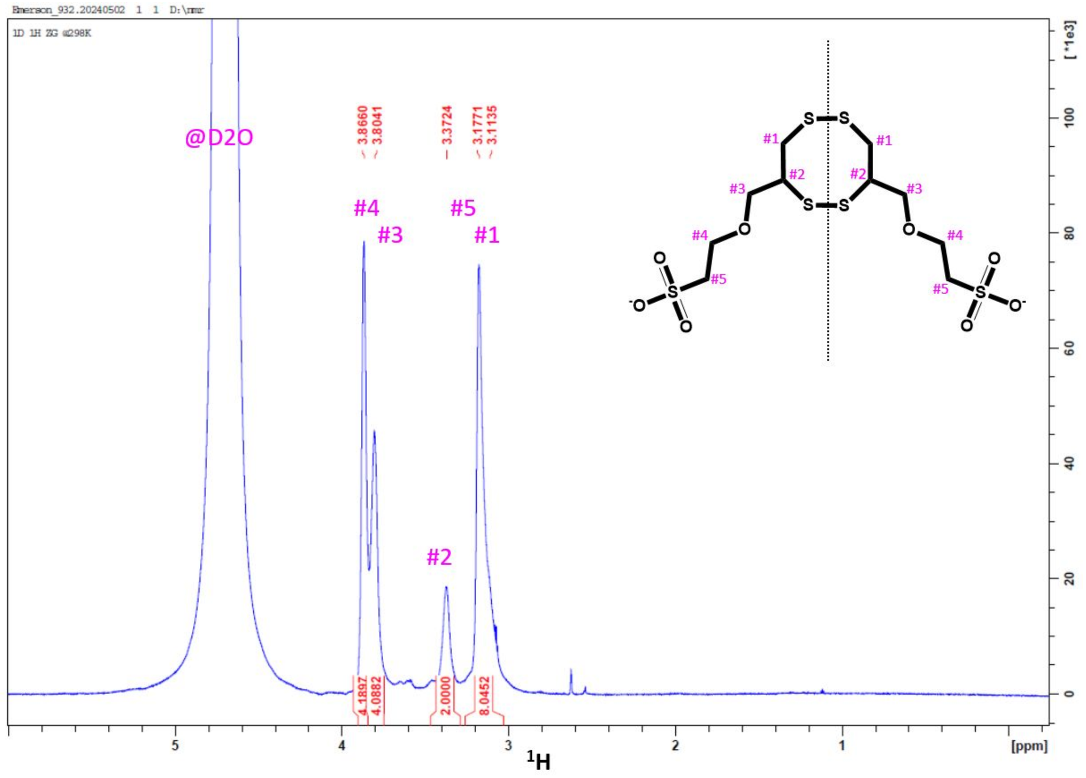

(B)

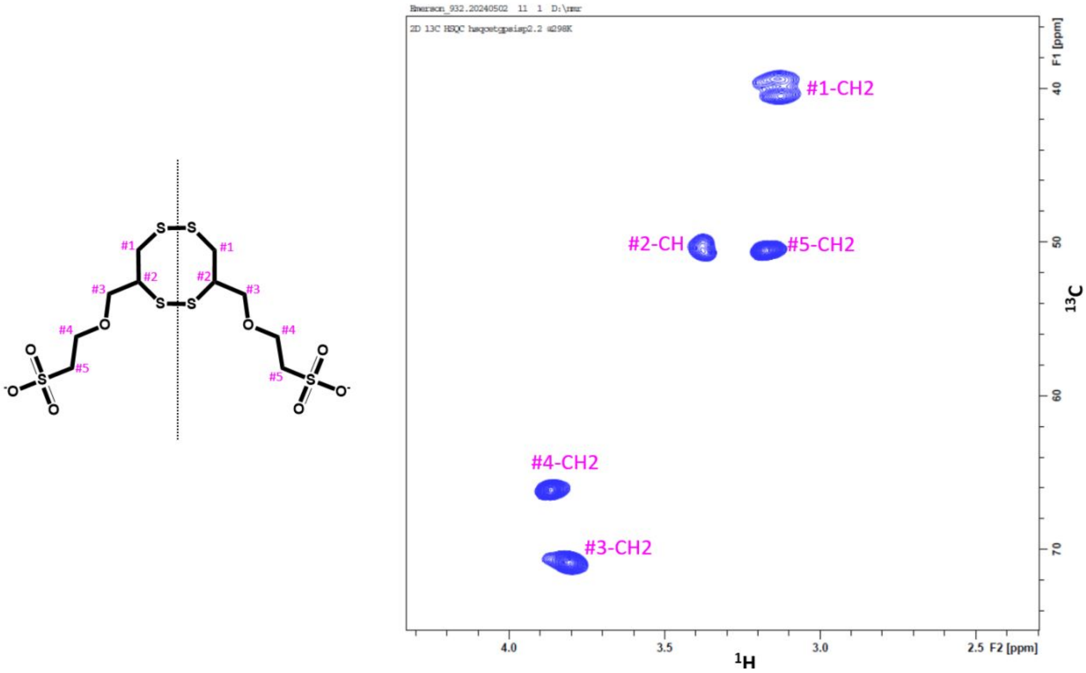

Figure S4. The 1D-^{1}H and 2D-^{1}H-^{13}C HSQC NMR spectra of TST1N-224. The chemical structure of **TST1N-224** was verified using NMR spectroscopy. NMR samples were prepared at a concentration of 5 mg/ml in D_2O, and the solutions were loaded into Shigemi NMR tubes for the experiments. The NMR spectra were recorded at 298 K using Bruker AVANCE III 600 MHz spectrometers equipped with a z-gradient TXI cryoprobe (Bruker, Karlsruhe, Germany). **(A)** The 1D-^{1}H spectrum of **TST1N-224** was acquired and analyzed to confirmed the chemical structure of **TST1N-224**. The protons were labeled and assigned (#1~#5). **(B)** The 2D-^{1}H-^{13}C NMR spectrum was acquired and analyzed to confirmed the chemical structure of **TST1N-224**. The protons were labeled and assigned (#1~#5). The acquired and analyzed 1D-^{1}H and 2D-^{1}H-^{13}C HSQC spectra confirmed the chemical structure of **TST1N-224**.

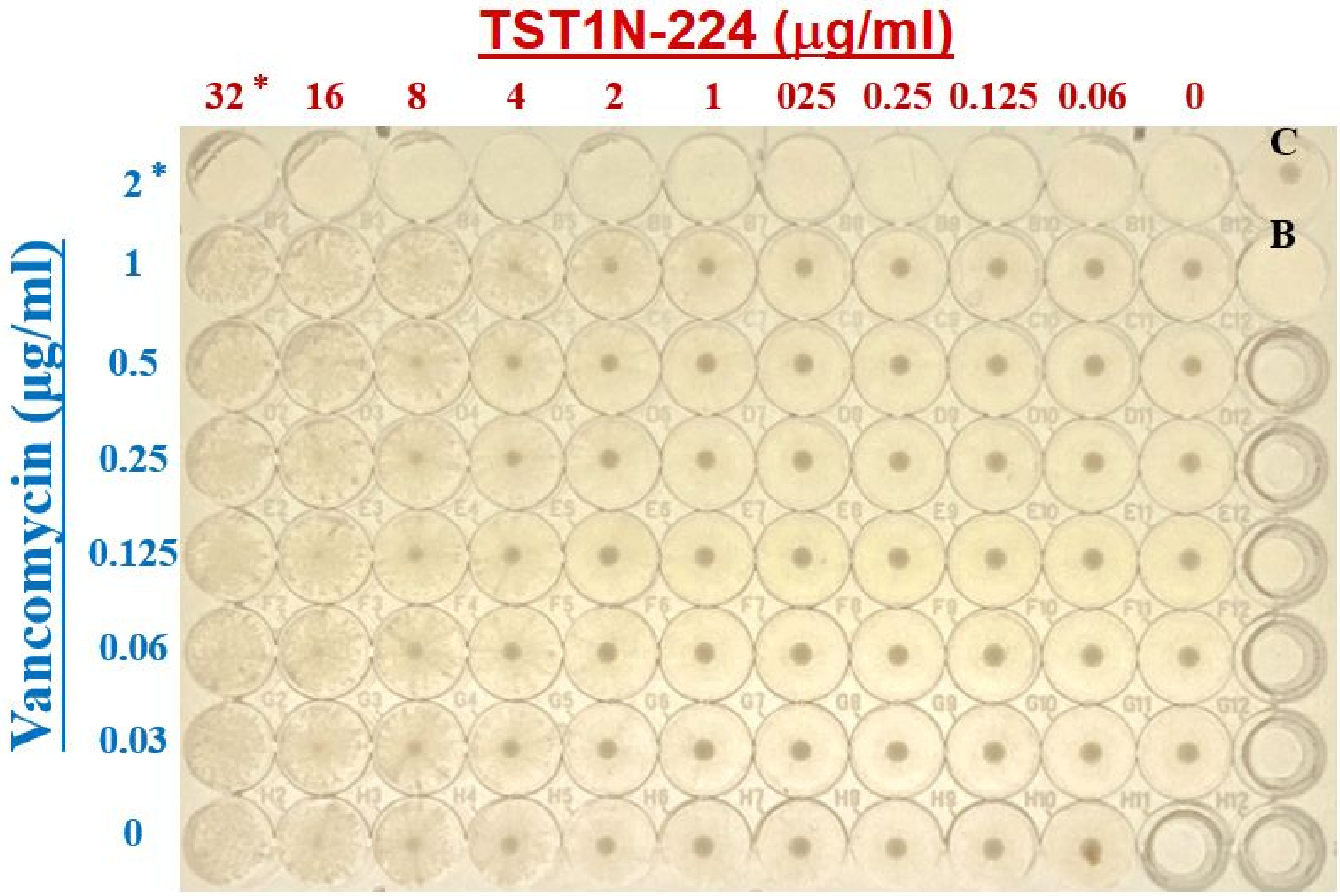

(C: control; B: Blank; Broth: MHB; Culture: 5×10^4 CFU/ml; * Denoting the MIC of compounds)

Figure S5. The FICI of TST1N-224 and vancomycin against VISA. The **TST1N-224** and vancomycin were combined to test their synergetic effect on the growth of VISA. The determined FICI was > 1, indicating the combination of **TST1N-224** and vancomycin showed no apparently synergetic effect to inhibit the growth of VISA.

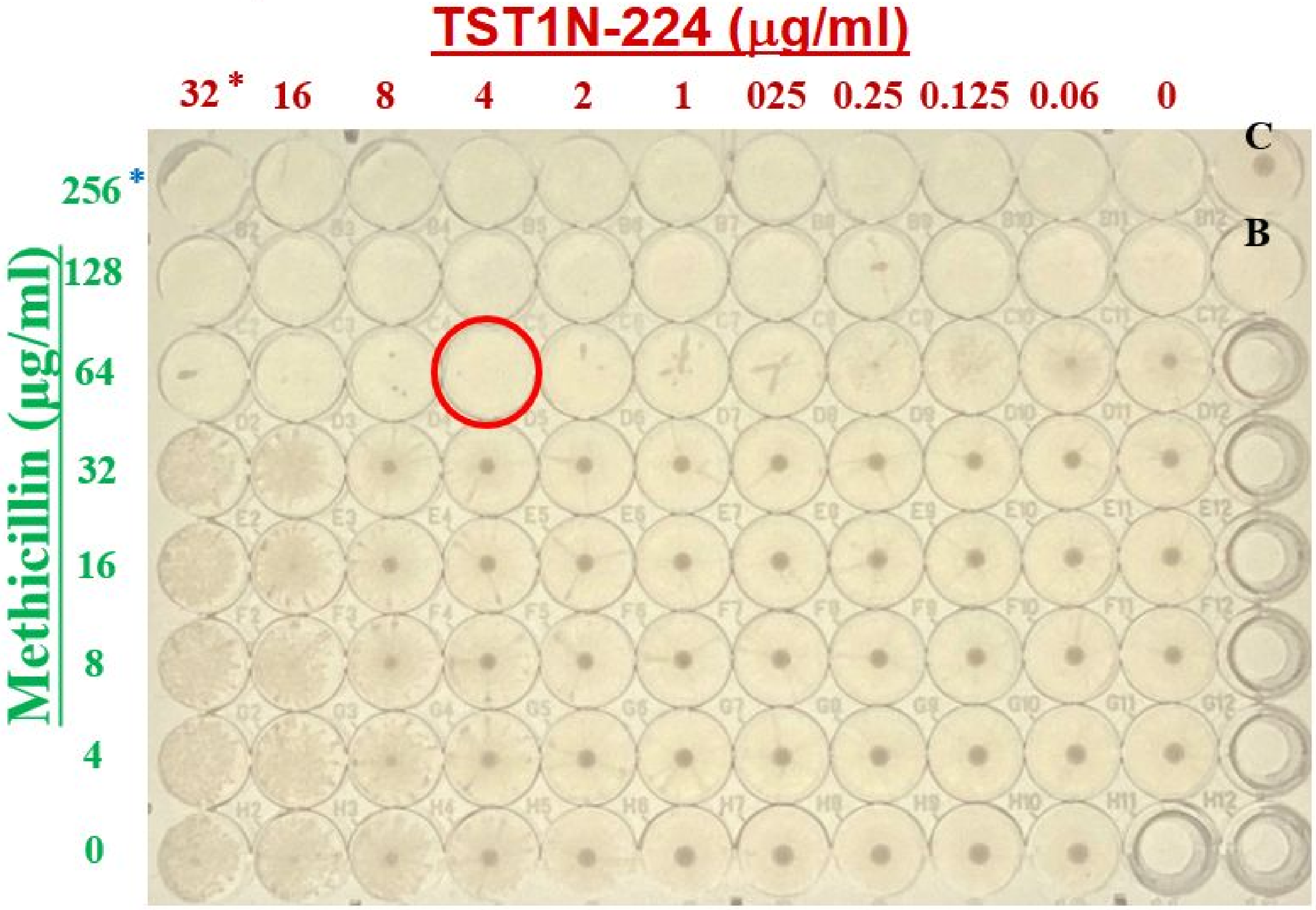

(C: control; B: Blank; Broth: MHB; Culture: 5 × 10^4 CFU/ml; * Denoting the MIC of compounds)

Figure S6. The FICI of TST1N-224 and methicillin against VISA. The **TST1N-224** and methicillin were combined to test their synergetic effect on the growth of VISA. The determined FICI was 0.675, indicating the combination of **TST1N-224** and methicillin showed apparently synergetic effect to inhibit the growth of VISA.

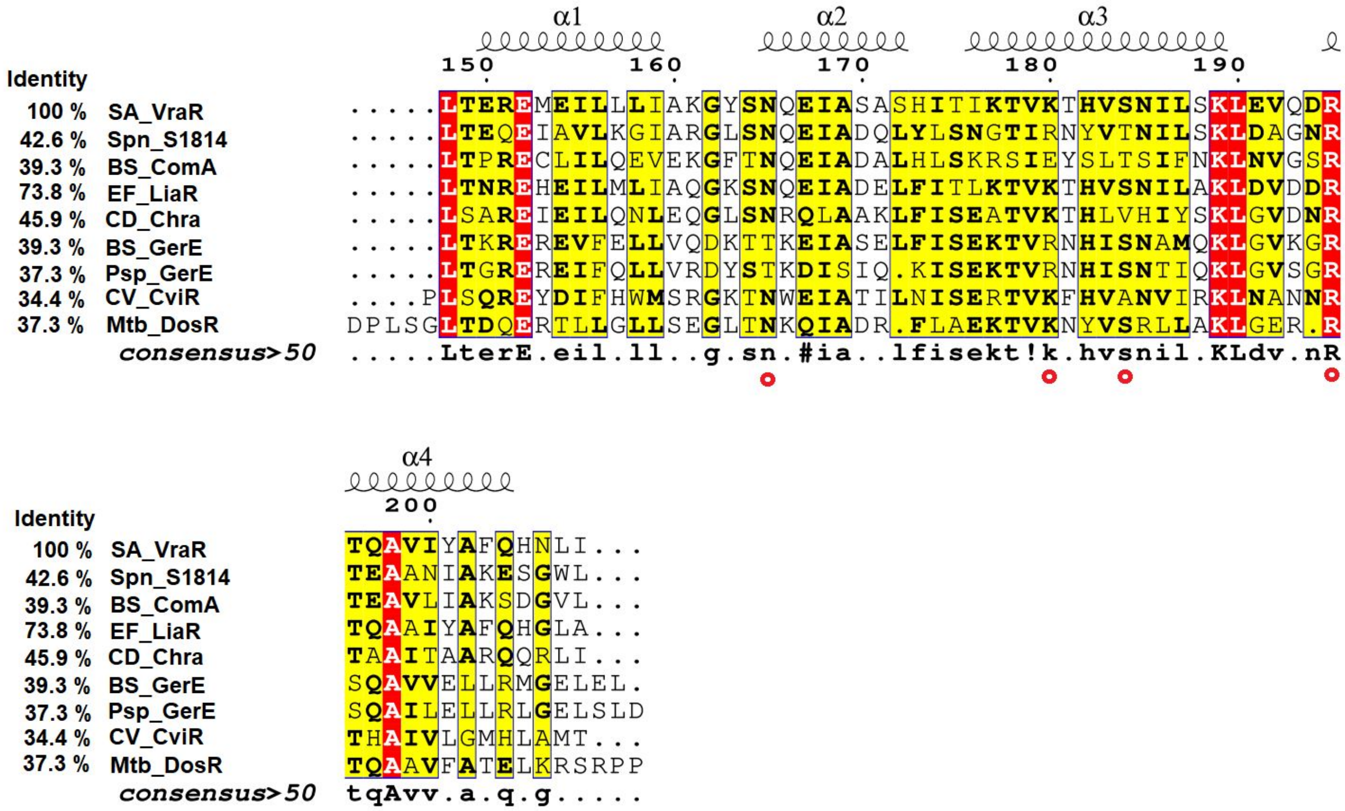

Figure S7. Multiple sequence alignment among VraRC and RRs of other bacterial species. The multiple sequence alignment of VraRC and RRs of other organisms was conducted using Clustal-omega[8] and presented by ESPript - http://espript.ibcp.fr. The red circles denote the key residues of VraRC interacting with **TST1N-224**. The abbreviation of names of bacteria are as follows. **SA**: *Staphylococcus aureus*; **Spn**: *Streptococcus pneumoniae*; **BS**: *Bacillus subtilis*; **EF**: *Enterococcus faecalis*; **CD**: *Corynebacterium diphtheriae*; **Psp**: *Paenisporosarcina sp.*; **CV:** *Chromobacterium violaceum*; **Mtb**: *Mycobacterium tuberculosis*.

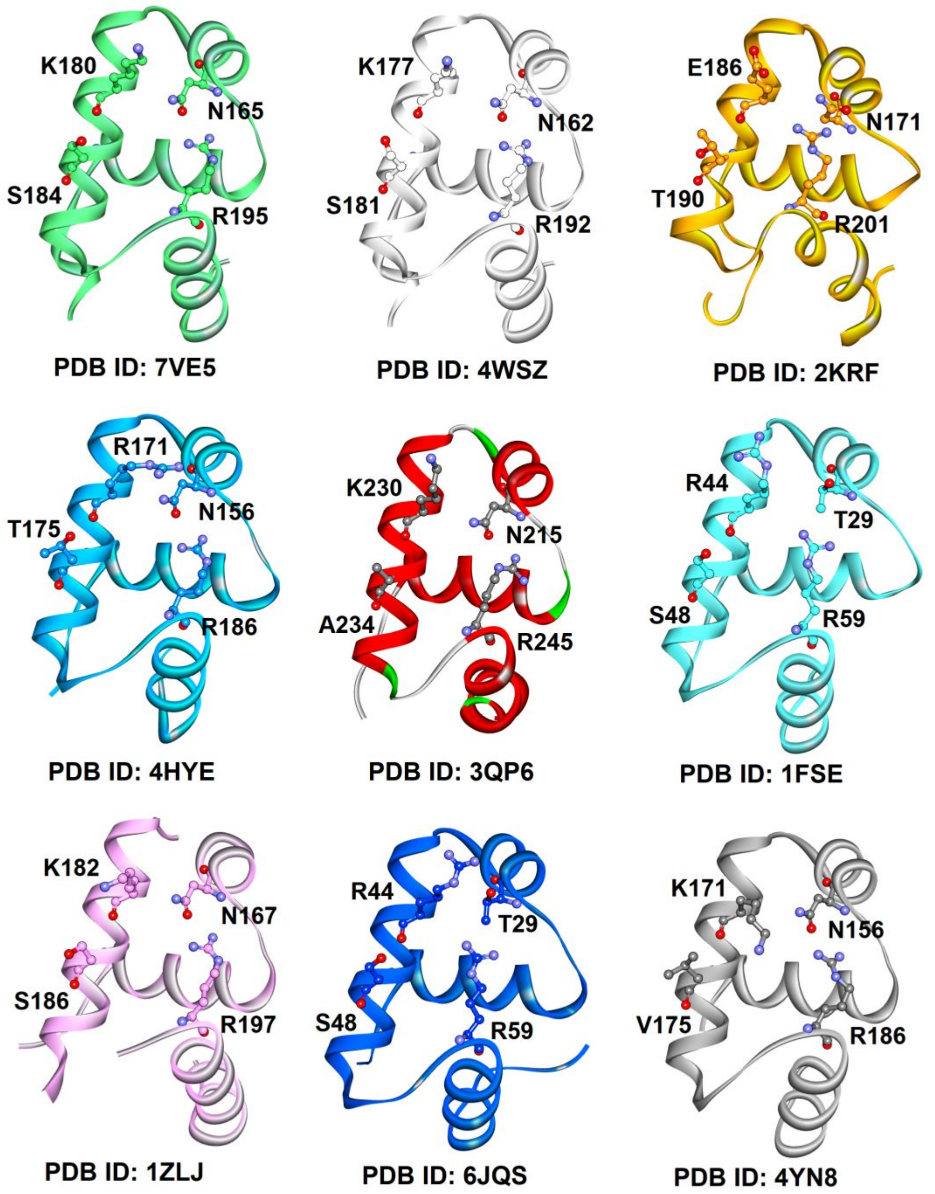

Figure S8. Structure conservations among VraRC and RRs of other bacterial species. The **TST1N-224** binding site (showed in sticks) is conserved among SA_VraR (PDB ID: 7VE5), EF_LiaR (PDB ID: 4WSZ), BS_ComA (PDB ID: 2KRF), Spn_S1814 (PDB ID: 4HEY), CV_CviR (PDB ID: 3QP6), BS_GerE (PDB ID: 1FSE), Mtb_DosR (PDB ID: 1ZLJ), Psp_GerE (PDB ID: 6JQS), and CD_ChrA (PDB ID: 4YN8). The abbreviation of names of bacteria are as follows. **SA**: *Staphylococcus aureus*; **Spn**: *Streptococcus pneumoniae*; **BS**: *Bacillus subtilis*; **EF**: *Enterococcus faecalis*; **CD**: *Corynebacterium diphtheriae*; **Psp**: *Paenisporosarcina sp*.; **CV:** *Chromobacterium violaceum*; **Mtb**: *Mycobacterium tuberculosis*.
